# Supplementary figures and images for: Afrotropical sand fly-host plant relationships in a leishmaniasis endemic area, Kenya
Source: PLoS Negl Trop Dis. 2021 Feb 8;15(2):e0009041. doi: 10.1371/journal.pntd.0009041 (PMC7895382; doi:10.1371/journal.pntd.0009041)

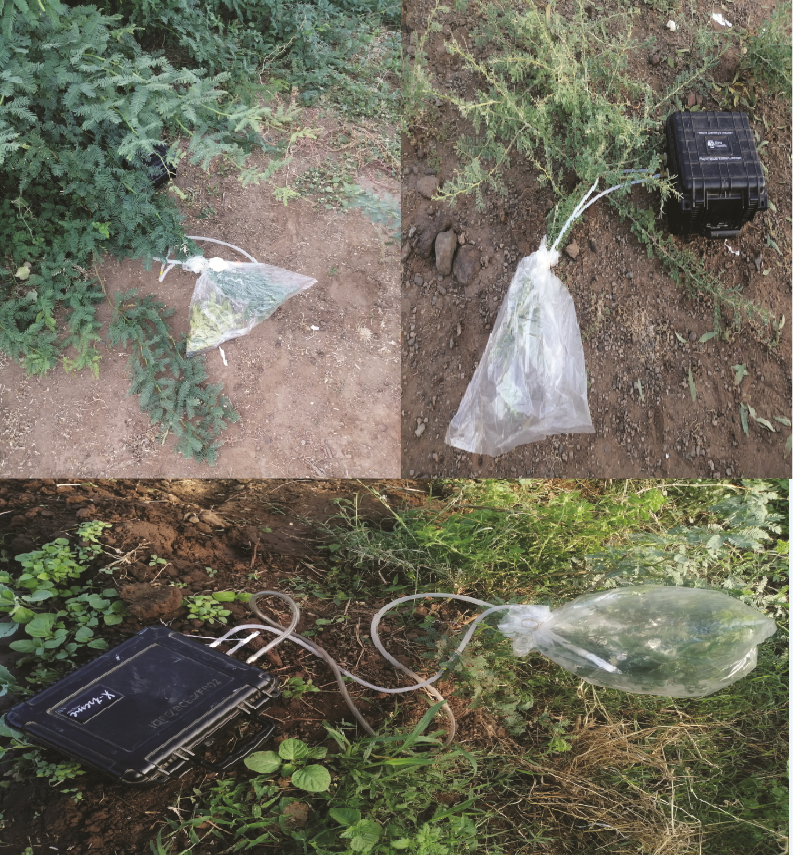

Supplement: S1 Fig — (TIF) [file pntd.0009041.s001.tif]
