# Supplementary material for: Afrotropical sand fly-host plant relationships in a leishmaniasis endemic area, Kenya
Source: PLoS Negl Trop Dis. 2021 Feb 8;15(2):e0009041. doi: 10.1371/journal.pntd.0009041 (PMC7895382; doi:10.1371/journal.pntd.0009041)
Supplement: S1 Table — (DOCX) [file pntd.0009041.s002.docx]

| Sand fly species or plant | **Sex** | **Location** | **Scientific name of Plant** | **Common name** | **Type of plant** | **Family** |
| --- | --- | --- | --- | --- | --- | --- |
| *P. duboscqi* | Female | Outdoor | *Vachellia sp* | *Acacia* sp | Tree | Fabaceae |
| *P. duboscqi* | Male | Outdoor | *Vachellia tortilis* | *Acacia tortilis* | Tree | Fabaceae |
| *P. duboscqi* | Female | Outdoor | *Senegalia laeta* | *Acacia laeta* | Shrub or small tree | Fabaceae |
| *P. duboscqi* | Female | Outdoor | *Vachellia tortilis* | *Acacia tortilis* | Tree | Fabaceae |
| *S. schwetzi* | Female | Indoor | *Senegalia laeta* | *Acacia laeta* | Shrub or small tree | Fabaceae |
| *S. schwetzi* | Male | Indoor | *Musa ABB Group* | - | Tree | Musaceae |
| *S. schwetzi* | Female | Indoor | *Senegalia laeta* | *Acacia laeta* | Shrub or small tree | Fabaceae |
| *S. schwetzi* | Male | Indoor | *Senegalia laeta* | *Acacia laeta* | Shrub or small tree | Fabaceae |
| *S. schwetzi* | Female | Indoor | *Musa ABB Group* | - | Tree | Musaceae |
| *S. schwetzi* | Female | Indoor | *Musa ABB Group* | - | Tree | Musaceae |
| *S. schwetzi* | Female | Indoor | *Vachellia tortilis* | *Acacia tortilis* | Tree | Fabaceae |
| *S. schwetzi* | Male | Indoor | *Solanum lycopersicum* | Tomato plant | Small tree | Solanaceae |
| *S. schwetzi* | Female | Indoor | *Cenchrus purpureus* | Elephant grass | Grass | Poaceae |
| *S. schwetzi* | Male | Indoor | *Vachellia tortilis* | *Acacia tortilis* | Tree | Fabaceae |
| *S. schwetzi* | Female | Indoor | *Vachellia tortilis* | *Acacia tortilis* | Tree | Fabaceae |
| *S. antennata* | Female | Indoor | *Vachellia tortilis* | *Acacia tortilis* | Tree | Fabaceae |
| *S. africana africana* | Male | Indoor | *Entada africana* | Tawatsa | Small tree | Fabaceae |
| *S. schwetzi* | Male | Indoor | *Prosopis juliflora* | Mesquite | Shrub or small tree | Fabaceae |
| *S. squamipleuris* | Female | Indoor | *Senegalia laeta* | *Acacia laeta* | Shrub or small tree | Fabaceae |
| *P. duboscqi* | Male | Outdoor | *Vachellia tortilis* | *Acacia tortilis* | Tree | Fabaceae |
| *S. schwetzi* | Male | Indoor | *Laurus nobilis* | Bay laurel | Tree or large shrub | Lauraceae |
| *S. schwetzi* | Male | Indoor | *Vachellia tortilis* | *Acacia tortilis* | Tree | Fabaceae |
| *S. schwetzi* | Female | Outdoor | *Eleusine indica* | Indian goosegrass | Small annual grass | Poaceae |
| *S. schwetzi* | Female | Outdoor | *Vachellia tortilis* | *Acacia tortilis* | Tree | Fabaceae |
| *S. schwetzi* | Female | Outdoor | *Senegalia laeta* | *Acacia laeta* | Shrub or small tree | Fabaceae |
| *S. schwetzi* | Male | Outdoor | *Faidherbia albida* | White acacia | Tree | [Fabaceae](http://powo.science.kew.org/taxon/urn:lsid:ipni.org:names:30000147-2) |
| *S. schwetzi* | Female | Outdoor | *Sporobolus vaginiflorus* | Poverty grass | Grass | Poaceae |
| *S. schwetzi* | Male | Outdoor | *Musa rosea* | Wild banana | Tree | Musaceae |
| *P. martini* | Male | Outdoor | *Vachellia tortilis* | *Acacia tortilis* | Tree | Fabaceae |
| *S. schwetzi* | Female | Outdoor | *Chloris virgata* | Feather finger grass | Grass | Poaceae |
| *S. schwetzi* | Male | Outdoor | [*Prosopis juliflora*](https://blast.ncbi.nlm.nih.gov/Blast.cgi#alnHdr_557798777) | Mesquite | Perennial shrub or tree | Fabaceae |
| *P. martini* | Female | Outdoor | *Gynerium sagittatum* | Uva Grass | Grass | Poaceae |
| *S. antennata* | Female | Outdoor | *Vachellia tortilis* | *Acacia tortilis* | Tree | Fabaceae |
| *S. schwetzi* | Female | Outdoor | *Senegalia laeta* | *Acacia laeta* | Shrub or small tree | Fabaceae |
| *S. schwetzi* | Female | Outdoor | *Vachellia tortilis* | *Acacia tortilis* | Tree | Fabaceae |
| *S. schwetzi* | Female | Outdoor | *Vachellia tortilis* | *Acacia tortilis* | Tree | Fabaceae |
| *S. schwetzi* | Male | Outdoor | *Prosopis juliflora* | Mesquite | Shrub or small tree | Fabaceae |
| *S. schwetzi* | Female | Outdoor | *Vachellia nilotica* | Gum arabic tree | Tree | Fabaceae |
| *P. martini* | Male | Outdoor | *Vachellia tortilis* | *Acacia tortilis* | Tree | Fabaceae |
| *P. martini* | Male | Outdoor | *Vachellia nilotica* | Gum arabic tree | Tree | Fabaceae |
| Plant |  |  | *Vachellia tortilis* | *Acacia tortilis* | Tree | Fabaceae |
| Plant |  |  | *Senegalia laeta* | *Acacia laeta* | Tree | Fabaceae |
| Plant |  |  | *Prosopis juliflora* | Mesquite | Tree | Fabaceae |
| Plant |  |  | *Vachellia tortilis* | *Acacia tortilis* | Tree | Fabaceae |
| Plant |  |  | *Vachellia tortilis* | *Acacia tortilis* | Tree | Fabaceae |
| Plant |  |  | *Vachellia tortilis* | *Acacia tortilis* | Tree | Fabaceae |
| Plant |  |  | *Vachellia sp* | *Acacia* sp | Tree | Fabaceae |

-, no known common names
