# Supplementary material for: Afrotropical sand fly-host plant relationships in a leishmaniasis endemic area, Kenya
Source: PLoS Negl Trop Dis. 2021 Feb 8;15(2):e0009041. doi: 10.1371/journal.pntd.0009041 (PMC7895382; doi:10.1371/journal.pntd.0009041)
Supplement: Attachment — Submitted filename: PNTD-D-20-01826_reviewer (1)_MFO.pdf [file pntd.0009041.s003.pdf]

PLOS Neglected Tropical Diseases

Afrotropical sand fly-host plant interactions in a leishmaniasis endemic area, Kenya

--Manuscript Draft--

**Manuscript Number:**	PNTD-D-20-01826
**Full Title:**	Afrotropical sand fly-host plant interactions in a leishmaniasis endemic area, Kenya
**Short Title:**	Afrotropical sand fly-host plant interactions
**Article Type:**	Research Article
**Keywords:**	Plant tissue feeding, Phlebotomus duboscqi, Phlebotomus martini, DNA barcoding, Semiochemical, Kenya
**Abstract:**	The bioecology of phlebotomine sand flies is intimately linked to the utilization of environmental resources including plant feeding. However, plant feeding behavior of sand flies remains largely understudied for Afrotropical species. Here, using a combination of biochemical, molecular, and chemical approaches, we decipher specific plant-feeding associations in field-collected sand flies from a dry ecology endemic for leishmaniasis in Kenya. Cold-anthrone test indicative of recent plant feeding showed that fructose positivity rates were similar in both sand fly sexes and between those sampled indoors and outdoors. Analysis of derived sequences of the ribulose bisphosphate carboxylase gene large subunit (rbcL) from fructose-positive specimens implicated mainly Acacia plants in the family Fabaceae (73%) as those readily foraged on by both sexes of Phlebotomus and Sergentomyia. Chemical analysis by high performance liquid chromatography detected fructose as the most common sugar in sand flies and leaves of selected plant species in the Fabaceae family. Analysis of similarities (ANOSIM) of the headspace volatile profiles of selected Fabaceae plants identified benzyl alcohol, (Z)-linalool oxide, (E)-β-ocimene, p-cymene, p-cresol, and m-cresol, as discriminating compounds between the plant volatiles. These results indicate selective sand fly plant feeding and suggest that the discriminating volatile organic compounds could be exploited in attractive toxic sugar- and odor- bait technologies for control strategies.
**Additional Information:**	
**Question**	**Response**
**Financial Disclosure**<br><br>Enter a financial disclosure statement that describes the sources of funding for the work included in this submission. Review the submission guidelines for detailed requirements. View published research articles from *PLOS NTDs* for specific examples.<br><br>This statement is required for submission and **will appear in the published article** if the submission is accepted. Please make sure it is accurate.	The funders had no role in study design, data collection and analysis, decision to publish, or preparation of the manuscript.

Powered by Editorial Manager® and ProduXion Manager® from Aries Systems Corporation

**Unfunded studies**<br><br>Enter: *The author(s) received no specific funding for this work.*<br><br>**Funded studies**<br><br>Enter a statement with the following details:<br>• Initials of the authors who received each award<br>• Grant numbers awarded to each author<br>• The full name of each funder<br>• URL of each funder website<br>• Did the sponsors or funders play any role in the study design, data collection and analysis, decision to publish, or preparation of the manuscript?<br>• **NO** - Include this sentence at the end of your statement: *The funders had no role in study design, data collection and analysis, decision to publish, or preparation of the manuscript.*<br>• **YES** - Specify the role(s) played.<br><br>* typeset	
**Competing Interests**<br><br>Use the instructions below to enter a competing interest statement for this submission. On behalf of all authors, disclose any competing interests that could be perceived to bias this work—acknowledging all financial support and any other relevant financial or non-financial competing interests.<br><br>This statement **will appear in the published article** if the submission is accepted. Please make sure it is accurate. View published research articles from *PLOS NTDs* for specific examples.	The authors have declared that no competing interests exist.

**NO authors have competing interests**<br><br>Enter: *The authors have declared that no competing interests exist.*<br><br>**Authors with competing interests**<br><br>Enter competing interest details beginning with this statement:<br><br>*I have read the journal's policy and the authors of this manuscript have the following competing interests: [insert competing interests here]*<br><br>* typeset	
**Data Availability**<br><br>Authors are required to make all data underlying the findings described fully available, without restriction, and from the time of publication. PLOS allows rare exceptions to address legal and ethical concerns. See the PLOS Data Policy and FAQ for detailed information.<br><br>A Data Availability Statement describing where the data can be found is required at submission. Your answers to this question constitute the Data Availability Statement and **will be published in the article**, if accepted.	Yes - all data are fully available without restriction

**Important:** Stating 'data available on request from the author' is not sufficient. If your data are only available upon request, select 'No' for the first question and explain your exceptional situation in the text box.<br><br>Do the authors confirm that all data underlying the findings described in their manuscript are fully available without restriction?	
**Describe where the data may be found in full sentences. If you are copying our sample text, replace any instances of XXX with the appropriate details.**<br><br>• If the data are **held or will be held in a public repository**, include URLs, accession numbers or DOIs. If this information will only be available after acceptance, indicate this by ticking the box below. For example: *All XXX files are available from the XXX database (accession number(s) XXX, XXX.*).<br>• If the data are all contained **within the manuscript and/or Supporting Information files**, enter the following: *All relevant data are within the manuscript and its Supporting Information files.*<br>• If neither of these applies but you are able to provide **details of access elsewhere**, with or without limitations, please do so. For example:<br><br>*Data cannot be shared publicly because of [XXX]. Data are available from the XXX Institutional Data Access / Ethics Committee (contact via XXX) for researchers who meet the criteria for access to confidential data.*<br><br>*The data underlying the results presented in the study are available*	All relevant data are<br>within the paper and its Supporting Information

from (include the name of the third party and contact information or URL).<br>• This text is appropriate if the data are owned by a third party and authors do not have permission to share the data.<br><br>* typeset	
Additional data availability information:	

Manuscript

Click here to access/download;Manuscript;manuscript file Oct152020F.docx

Afrotropical sand fly-host plant interactions in a leishmaniasis endemic area, Kenya

Iman B. Hassaballa[1,2], Catherine L. Sole[2], Xavier Cheseto[1], Baldwyn Torto[1,2], David P. Tchouassi[1]*

[1]International Centre of Insect Physiology and Ecology, Nairobi, Kenya,

[2] Department of Zoology and Entomology, University of Pretoria, Pretoria, South Africa

* Corresponding author: dtchouassi@icipe.org; Tel: +254 20 8632000; Fax: +254 20 8632001

Abstract

The bioecology of phlebotomine sand flies is intimately linked to the utilization of environmental resources including plant feeding. However, plant feeding behavior of sand flies remains largely understudied for Afrotropical species. Here, using a combination of biochemical, molecular, and chemical approaches, we decipher specific plant-feeding associations in field-collected sand flies from a dry ecology endemic for leishmaniasis in Kenya. Cold-anthrone test indicative of recent plant feeding showed that fructose positivity rates were similar in both sand fly sexes and between those sampled indoors and outdoors. Analysis of derived sequences of the ribulose bisphosphate carboxylase gene large subunit (rbcL) from fructose-positive specimens implicated mainly *Acacia* plants in the family Fabaceae (73%) as those readily foraged on by both sexes of *Phlebotomus* and *Sergentomyia*. Chemical analysis by high performance liquid chromatography detected fructose as the most common sugar in sand flies and leaves of selected plant species in the Fabaceae family. Analysis of similarities (ANOSIM) of the headspace volatile profiles of selected Fabaceae plants identified benzyl alcohol, (*Z*)-linalool oxide, (*E*)-*β*-ocimene, *p*-cymene, *p*-cresol, and *m*-cresol, as discriminating compounds between the plant volatiles. These results indicate selective sand fly plant feeding and suggest that the discriminating volatile organic compounds could be exploited in attractive toxic sugar- and odor- bait technologies control strategies.

Author summary

Plant feeding as an essential resource of sand flies, primary vectors of leishmania parasites, is largely understudied for Afrotropical species. Here, we combined field ecology, biochemical, molecular and chemical approaches, to decipher plant feeding associations in field-collected sand flies from a dry ecology endemic for leishmaniasis in Kenya revealing i) similar rates of plant feeding among sand fly sexes sampled from indoor and outdoor environments, ii) acacia plants in the family Fabaceae as those readily foraged on by sand fly species in *Phlebotomus* and *Sergentomyia*, iii) fructose as the common sugar in sand flies and leaves of selected plant species in the Fabaceae family, iv) compounds namely benzyl alcohol, (*Z*)-linalool oxide, (*E*)-*β*-ocimene, *p*-cymene, *p*-cresol, and *m*-cresol, as discriminating volatile organic compounds between volatiles of selected Fabaceae plants. The findings indicate selective sand fly plant feeding and suggest that the discriminating volatile organic compounds could be exploited in attractive toxic sugar- and odor-bait technologies for sand fly control.

Introduction

The sand fly-borne disease leishmaniasis, constitutes a public health problem in eastern Africa including Sudan, Ethiopia, Uganda, Kenya, and Somalia [1]. Leishmaniasis ranks among the priority list of zoonotic diseases for control in Kenya [2] where an estimated 4000 human cases of the visceral form (VL) of the disease occurs, with about 5 million at risk of infection [3,4]. Current control of leishmaniasis relies on prompt diagnosis and chemotherapeutic treatment. However, these approaches have not prevented the spread of the disease across Kenya. Visceral leishmaniasis has expanded in geographic range with frequent recent outbreaks as the cutaneous form (CL) of the disease [4]. New tools are therefore urgently needed with emphasis on disease prevention through effective management and control of the sand fly vectors.

Control of leishmaniasis by targeting the vectors can be achieved through an understanding of sand fly behavior, with prominent amongst them being plant feeding [5]. Plants serve as the primary source of energy for sand flies to support various biological functions including survival, fecundity, and dispersal. Male sand flies are exclusively feed on plants. Females obligately rely on plant derived-diets only sporadically feeding on blood [6]. As such, plant feeding offers an attractive target for developing surveillance and control strategies to limit human pathogen exposure risk. Examples include attractive toxic sugar bait (ATSB) [7–10] and an ‘attract and kill" strategy whereby a sugar solution as feeding substrate is laced with oral low risk toxins either as bait stations or applied on host plants [11,12].

Potential transmission blocking agents could be envisaged from the understanding of plants fed upon by sand flies in nature. This essential behavior exposes them to a range of plant produced metabolites which may affect their survival but also their vector competence to pathogens. Schlein and Jacobson [6] demonstrated that feeding on certain plants by the sand fly *Phlebotomus papatasi* (vector of CL) modulated the outcome of infection success with the parasite, *Leishmania major*. However, effective implementation of these intervention strategies requires precise information on plant feeding sources.

The use of sensitive molecular techniques such as polymerase chain reaction (PCR) and sequencing has improved our understanding of specific forage associations of sand flies and other arthropod disease vectors [13,14]. Using these approaches, Lima et al [15] demonstrated feeding preference by the Neotropical sand fly species *Lutzomyia longipalpis* on plants in the Fabaceae family. Recently, several leishmaniasis sand fly vectors were shown to prefer the plant *Cannabis sativa* for sugar feeding [16]. The specific plants that Afrotropical species of sand flies forage in nature are poorly understood. Specific sand fly-plant associations are reported in literature; for example, the occurrence of the sand fly vector *Phlebotomus orientalis* in habitats with *Acacia seyal* and *Balanites aegyptiaca* vegetation [17]. Nonetheless, understanding the ecologic functions underlying such relationships have not been fully described.

The objective of this study was to document the degree to which diverse wild-caught sand fly species including *Phlebotomus* vectors of leishmaniasis utilize plant feeding sugar resources in the natural setting, and explore the nutritional and semiochemical basis for host plant selection for feeding

Materials and Methods

Sample collection

Adult phlebotomine sand flies were collected in Rabai (0.45866° N, 35.9889° E), a rural community endemic for VL and CL located in the Marigat sub-County, Baringo County, Kenya (Fig 1). The area is a dry ecology characterized by numerous large termite mounds and sparse vegetation attributed to over-grazing by livestock [18,19]. The vegetation mainly consists of thorny and *Commiphora* bushes, *Acacia* trees, *Cactus* trees, and the invasive plant species *Prosopis juliflora* [19]. Sand flies were surveyed using Centers for Disease Control (CDC) miniature light traps (Model 512, John Hock Co., Gainesville, Florida, USA) in December 2018 with abundant sand flies. Traps were randomly deployed to collect night-active sand flies

from three habitat types: houses indoors and outdoors, around termite mounds and animal sheds. Sand flies were sorted, immediately frozen in liquid nitrogen and transported to the laboratory at *icipe* in Nairobi, where they were stored at –80 ^{o}C until further processing.

Sample preparation and identification

To remove any plant debris from the body of the sand fly, individual specimens were rinsed in 0.5% bleach solution for 1 min, and then twice in double distilled water (ddH$_2$O) each lasting 30 s as described previously in Nyasembe et al [13]. Sand flies were morphologically identified to species based on dissection and observation of the external genitalia (for males) and the head and spermatheca (for females) using published keys [20,21]. The remaining part of the body (abdomen) containing the crop was processed for biochemical and molecular analyses.

Determination of evidence of recent plant feeding in the field collection sand flies

Each sand fly abdomen containing the crop was macerated in 50 µL absolute ethanol using a sterilized pestle. An aliquot (25 µL) of the sand fly homogenate from each individual was placed in the wells of a flat-bottomed 96-well microplate, and tested for presence of fructose as evidence of recent plant feeding by the cold anthrone test [22] with modifications by Matheson et al [23]. Briefly, 200 µL anthrone solution (0.15% anthrone (Sigma -Aldrich) w/v in 71.7% sulphuric acid) was added to each well containing the homogenate and incubated for 60 min at room temperature (25°C). A change in color from yellow to green or blue is indicative of a positive test [22].

Plant DNA extraction from sand fly homogenate

DNA was extracted from the aliquot of homogenate (above) of fructose–positive samples using the ISOLATE II Plant DNA Kit (Bioline, London, UK) according to the manufacturer's instructions with a slight modification. This included extension of the incubation period with the lysis buffer PA1 and RNase by 4-6 hr and elution with Buffer PG by 10 min. The extracted DNA was stored at -20˚C until further processing.

PCR amplification, purification, and sequencing

A PCR target of 450 – 660 bp fragment of the the ribulose-1,5-bisphosphate carboxylase large subunit gene (rbcL) was used to identify plant DNA in sand flies, a widely used marker for plant barcoding studies. The primers used included rbcLaF (5'-ATGTCACCACAAACAGAGACTAAAGC-3') and rbcLaR (5'-

GTAAAATCAAGTCCACCRCG -3') [24,25]. Polymerase chain reaction (PCR) MyTaq DNA Polymerase Kit (Bioline, London, UK) in a total of 10 µL reaction volume containing 10µM each of the forward and reverse primers, 0.0625 U MyTaq DNA polymerase, 5X My Taq reaction buffer and 1-2µL of DNA template (1- 10 ng) was used for PCR using a Veriti 96-well Thermal Cycler (Singapore). Polymerase chain reaction (PCR) Thermal profile included initial denaturation at 94 °C for 4 min, followed by 35 cycles of 94 °C for 30 sec, 55 °C for 30 sec and 72 °C for 1 min, and final extension at 72 °C for 10 min. Amplicons were resolved on 1.5% agarose gel electrophoresis stained with ethidium bromide (Sigma-Aldrich, GmbH, Germany) against a 100 bp DNA ladder (HyperLadder, Bioline, London, UK). Polymerase chain reaction (PCR) water served as negative controls in all PCRs and DNA extracted directly from the tissue of selected plants as (described below) as positive controls. Polymerase chain reaction (PCR) products were purified using the ISOLATE II PCR and Gel Kit (Bioline, London, UK) or Exo/SAP-IT Kit **(**Affymetrix Inc., USA) as per the manufacturers' instructions. Purified products were outsourced for Sanger sequencing to generate both forward and reverse reads to Macrogen (Inc Europe Laboratory).

DNA sequence analysis

The forward and reverse sequences were cleaned as described in Tchouassi et al. (2014). Briefly, they were visually inspected, aligned and edited using MEGA v. 7 (Kumar et al., 2016). Consensus sequences for each sample were aligned using ClustalW in MEGA using default parameters. Each sequence was compared to a reference sequence in the GenBank database using Blanstn with the search option 'Highly similar sequences'. Sequences were assigned to specific plant species based on matches >98% [27].

Plant sample collection as a reference for identified sand fly host plants

The leaves of selected putatively identified plants based on sequence analysis were collected from the study site. The leaves were wrapped separately for each plant in newspaper in the field, labeled, and transported to the laboratory at *icipe* in Nairobi and dried at room temperature. 20 mg dry weight each of the grounded plants was similarly processed for DNA extraction, amplification and sequencing as described previously.

Fig 1. Map of the study site showing the environment where sand flies and plants were sampled from Rabai village in Baringo County, Kenya. The map was designed using ArcMap 10.2.2 with the ocean and lakes base layer derived from Natural Earth

(http://www.naturalearthdata.com/, a free GIS data source). The sample points were collected using a GPS gadget (garmin etrex 20, https://buy.garmin.com/en-US/US/p/518046), and the county boundaries for Kenya derived from Africa Open data (https://africaopendata.org/dataset/kenya-counties-shapefile, license Creative Commons).

Processing of plants and sand flies for sugar profiling

Leaves were sampled from selected Fabaceae plants including *Vachellia tortilis, Senegalia laeta*, *Vachellia nilotica* and *Prosopis juliflura* in the field which were identified as host plants for sand flies through sequence analysis (see Results section below). Also, two other acacia plants *Senegalia senegal, Vachellia elatior*, present in the study locality but their DNA not detected in the sand flies were sampled. The plant leaves were harvested and transported in wrapped newspaper to the lab at *icipe* in Nairobi, Kenya. They were separately air-dried under shade at room temperature and then ground to fine powder using an electric grinder (Retsch GmbH, Haan, Germany) and extracted following the method of Mokaya et al [28] with slight modifications. Briefly, 20 mg of each of the ground plant samples (leaves) was extracted in 1 ml (3:1 distilled deionized water: acetonitrile), vortexed for 10s, sonicated for 1 h, and centrifuged at 14,000 rpm for 5 min. The supernatant was filtered using qualitative filter paper (Whatman, circles, diam. 25 mm) and analyzed for sugars by High Performance Liquid Chromatography (HPLC) (described below). The plant materials were analyzed in triplicate, with each replicate collected from different plant samples. The abdomen containing the crop was dissected from wild caught sand flies and pooled (50/pool) by sex, extracted, and analyzed for sugars as described for the plants.

Chemical analysis of sugars in identified plants and sand flies

For sugar detection,10 µl of each plant and sand fly sample extract was analyzed by high performance liquid chromatography (HPLC) following previously established methods for sugars [28]. This was carried out on an Agilent HPLC system (Palo Alto, CA) equipped with a 1260 refractive index detector (RID) and photo diode array detector (wavelength set at 190−360 nm for UV and 360−700 nm for visible range). The column oven temperature was set at 30 °C with the following column parameters, 250mm × 4.6mm i.d., 5 µm, LC-NH2 column (Supelco, Bellefonte, PA, USA). The mobile phases consisted of an isocratic system water (A) and acetonitrile (B), (75:25 v/v). Samples were tested for presence of the following sugars: fructose, glucose, sucrose, galactose, maltose, xylose, trehalose and lactose, which were identified by comparing their retention times and co-injection with those of the authentic

samples. Serial dilutions of the authentic sugar standards (0.1–100 ng/µl) were analyzed also to generate a linear calibration curves which gave coefficient of determinations of R^2; fructose (0.9999), glucose (0.9998) and sucrose (0.9999). These regression equations were used for the external quantification of the different sugars found in the plants and sand fly samples, respectively.

Collection of volatiles from selected identified plants in the field

Headspace volatiles targeting the vegetative parts were collected from four of the identified host plants fed on by sand flies (~70% of samples analyzed) based on plant DNA detection namely, *V. tortilis, S. laeta*, *V. nilotica* and *P. juliflora* (see results section). This was carried out *in situ* in their natural habitats at the study site, using portable field pumps (Analytical Research System, Gainesville, Florida, USA). Odors from the four plants were collected by enclosing a vegetative part in an airtight oven bag (Reynolds, Richmond, VA, USA) (S1 Fig) by passing charcoal-filtered air at a flow rate of 350 ml/min on to two Super-Q adsorbents (30 mg, Analytical Research System, Gainesville, Florida, USA) for each replicate substrate. For all the plant species, volatiles were collected for 12 hr (06:00 -18:00) during the day and 12 hr (18:00 - 06:00) at night and replicated three to four times for each plant using a different plant. The same procedure was applied for the control, comprising volatiles from blank oven bags in the same habitat of the target plant. The Super-Q traps were each eluted with dichloromethane (DCM) (200 µl) (GC-grade, Sigma Aldrich, Gilling-ham, UK) and analyzed using Gas chromatography/mass spectrometry (GC-MS).

Chemical analysis of volatiles from selected identified plants

Plant volatile extracts were analyzed using coupled gas chromatography/mass spectrometry (GC-MS). An aliquot (1 µl) of DCM volatile extract of each sample and a blank were injected into a gas chromatograph coupled to a mass spectrometer (GC-MS) in a splitless injection mode. The GC was equipped with an HP-5 column (30 m x 0.25 mm ID x 0.25 µm film thickness) with helium as the carrier gas at a flow rate of 1.2 ml/min. The oven temperature was held at 35 °C for 5 min, then programmed to increase at 10 °C/min to 280 °C and was maintained at this temperature for 10.5 min. The mass selective detector was maintained at ion source temperature of 230 °C and a quadrupole temperature of 180 °C. Electron impact (EI) mass spectra was obtained at the acceleration energy of 70 eV. Fragment ions were analyzed over 40–550 *m/z* mass range in the full scan mode. The filament delay time was set at 3.3 min.

The compounds were identified by comparison of mass spectrometric data and retention times with those of authentic samples and reference spectra published by library–MS databases: Adams2, Chemecol and NIST (0.5, 0.8 and 11). Compounds present in controls were excluded from compositional profiles in each sample. Furthermore, identification of the VOCs in each plant was achieved based on their retention indices (RI). RI was determined with reference to a homologous series of normal alkanes C_8-C_{23} and calculated using the equation below as described by Van den Dool and Kratz [29] and comparison with published literature [30–32].

$$\mathbf{RIx=100\, n_0 + 100\, (R_Tx - R_Tn_1) / (R_Tn_1 - R_Tn_0)}$$

With:

x = the name of the target compound

n_1 = n-alkane $C_{n1}H_{2n1+2}$ directly eluting before x

n_0 = n-alkane $C_{no}H_{2nO+2}$ directly eluting after x

R_T = retention time

RI = retention index

Chemicals

The chemicals used for HPLC including D-(+)-xylose, 99%, D-(+)-galactose 99%, D-(-)-fructose 99%, sucrose 99.5%, maltose 99%, D-(+)-glucose 99.5%, D-(+)- trehalose 99%, D-lactose 99.5%) and acetonitrile (ACN) (HPLC grade) were purchased from Sigma-Aldrich, St. Louis, Missouri, United States. Chemicals used in the GC-MS analysis including hexanal, heptanal, benzaldehyde, octanal, nonanal, decanal, 6-methyl-5-hepten-2-one, acetophenone, *α*-pinene, *p*-cymene, sabinene, *β*-pinene, (*E*)-*β*-ocimene, (*Z*)-linalool oxide (furanoid), (*Z*)-linalool oxide (pyranoid), *α*-cedrene, octanol, 1-octen-3-ol, phytol, benzyl alcohol, *m*-cresol, *p*-cresol, indole, methyl salicylate and standard n-alkanes solution were purchased from Sigma Aldrich All the chemicals were >97% purity.

Statistical analysis

The proportion of fructose positive sand flies sampled outdoor (termite mounds + animal shed) and indoor (houses), was subjected to a Chi- squared test to compare proportions. The amount of sugar type in the different plants and sand fly crop was expressed as mean ± standard error based on 3 replicate runs. Analysis of variance (ANOVA) was carried out to compare the amounts for each sugar detected between the plants and sand fly species followed by mean separation using Tukey's HSD (Honest Significant Test) after checking for normality using Shapiro-Wilk test. All analyses were performed in R version 3.6.3 [33] at 95% significance

level. Similarity percentages (SIMPER) analysis was performed on peak areas of volatile compounds identified by GC-MS to determine the relative contribution of different compounds to the dissimilarity among volatiles of the different plants. The output was visualized using the non-metric multidimensional scaling approach. One-way analysis of similarities (ANOSIM) using Bray–Curtis dissimilarity matrix was performed to compare the chemical profiles of different plants volatiles using Past 3 free software [34]. The frequency of plant meal sources for each sand fly regardless of sex was classed into families and visualized using the heatmap, generated using Past 3 free software [34].

Ethical statement

Approval for the study was sought from the Scientific Ethics and Review Committee of the Kenya Medical Research Institute (SERU-KEMRI) (Protocol number: 3312). In addition, verbal consent was obtained from the chief of Rabai village and heads of households selected for sampling sand flies from inside and outside houses.

Results

Evidence of recent plant feeding in wild-caught sand fly species

Six hundred sand flies were tested for fructose by the cold anthrone test. These comprised 300 sampled indoors in houses and 300 outdoors from both termite mounds and animal sheds. Overall, 38.7% (116/300) were fructose positive indoors compared to 35.7% (107/300) outdoors. The difference was not statistically significant ($\chi 2 = 0.36$, $df = 1$, $P = 0.5$). Fructose positive rate of 44.7% (67/150) for females and 32.7% (49/150) for males significantly differ for collections indoors ($\chi 2 = 2.8$, $df = 1$, $P < 0.09$). Similarly, for outdoor collections, 33.3% (50/150) males and 38% (57/150) females tested positive for fructose and the difference was not statistically significant ($\chi 2 = 0.46$, $df = 1$, $P = 0.5$). The data by species are presented in (Table 1).

Table 1. Sugar feeding status of wild-caught phelobotomine sand fly species in Rabai village

	Indoor (No. fructose positive (No. tested)		Outdoor (No. fructose positive (No. tested)	
Sand fly species	Male	Female	Male	Female
P. duboscqi	0(0)	0(0)	2(7)	5(7)
P. martini	0(5)	1(5)	7(45)	4(45)

S. antennata	13(98)	24(98)	1(25)	6(25)
S. africana africana	9(58)	7(58)	0(7)	0(7)
S. clydei	0(3)	0(3)	1(15)	1(15)
S. schwetzi	27(131)	32(131)	39(179)	36(179)
S. squamiplura	0(5)	3(5)	0(22)	5(22)
Total	49(150)	67(150)	50(150)	57(150)

Plant DNA amplification from fructose-positive sand flies

A subset of fructose positive sand flies (n=221) were processed by PCR of which 83 amplified for rbcLa (Table 2). Forty of the rbcL amplicons were successfully sequenced (Table 2). Polymerase chain reaction (PCR) success rate and sequencing varied between the different sand fly species analyzed. The sequenced fragment sizes ranged between 450 and 660 bp.

Table 2. Variable success rates in amplifying and sequencing plant DNA in different sand fly species

Sand fly species	N =fructose positive	No. amplified (No. sequenced successfully)
P. duboscqi	7	5(5)
P. martini	12	10(4)
S. africana africana	16	6(1)
S. antennata	44	10(2)
S. schwetzi	134	50(27)
S. squamipleuris	8	2(1)
**Overall**	**221**	**83(40)**

N = number of sandflies from which plant DNA were extracted

Afro-tropical sand flies preferentially feed on plants in the Fabaceae family

Analysis of the 40 derived rbcL sequences from the sand flies implicated 16 plant species as feeding sources belonging to 5 families *viz*: Fabaceae, Musaceae, Solanaceae, Poaceae, Lauraceae (Fig 2; Table 3). The plants identified for males as candidate sugar sources belong to the families Fabaceae, Musaceae, Solanaceae and Lauraceae, whereas for females they were Fabaceae, Poaceae, and Musaceae. As such, the key plant families associated with both sexes included Fabaceae, Musaceae and Poaceae. Most of the plants identified were acacias belonging to the family Fabaceae and represented by at least 4 species *V. tortilis*, *S. laeta*, *V. nilotica* and *Faidherbia albida* (Table 3). Analysis of the plant species profiles of the Fabaceae

detected showed that sand flies of both sexes had predominantly fed on *V. tortilis*. The two leishmaniasis vectors analyzed *P. duboscqi* and *P. martini* exclusively or predominantly had fed on acacia plants belonging the family Fabaceae (Table 3). A minor representation of food crops and herbs or grasses were implicated as sand fly feeding sources. Only 3 specimens of *S. schwetzi* had fed on the invasive weed species *P. juliflora* (Fabaceae).

Table 3. The composition of host plants identified from sand fly species in Rabai village, Baringo County, Kenya

	Sample ID	Sand fly Species and plants	Sex	Scientific name of Plant	Common name	Type of plant	Family	Query cover	Per. Identity	Accession
1	31A	*P. duboscqi*	Female	*Vachellia sp*	*Acacia* sp	Tree	Fabaceae	100%	100%	MN216486.1
2	40A	*P. duboscqi*	Male	*Vachellia tortilis*	*Acacia tortilis*	Tree	Fabaceae	100%	99.29%	MN216557.1
3	41A	*P. duboscqi*	Female	*Senegalia laeta*	*Acacia laeta*	Shrub or small tree	Fabaceae	100%	99.47	MN216705.1
4	42A	*P. duboscqi*	Female	*Vachellia tortilis*	*Acacia tortilis*	Tree	Fabaceae	100%	99.47%	MN216557.1
5	62H	*S. schwetzi*	Female	*Senegalia laeta*	*Acacia laeta*	Shrub or small tree	Fabaceae	100%	99.82%	MN216705.1
6	209H	*S. schwetzi*	Male	*Musa ABB Group*	N/A	Tree	Musaceae	100%	99.06%	KR073285.1
7	211H	*S. schwetzi*	Female	*Senegalia laeta*	*Acacia laeta*	Shrub or small tree	Fabaceae	100%	99.29%	MN216705.1
8	215H	*S. schwetzi*	Male	*Senegalia laeta*	*Acacia laeta*	Shrub or small tree	Fabaceae	100%	100%	MN216705.1
9	222H	*S. schwetzi*	Female	*Musa ABB Group*	N/A	Tree	Musaceae	100%	100%	KR073285.1
10	228H	*S. schwetzi*	Female	*Musa ABB Group*	N/A	Tree	Musaceae	100%	100%	KR073285.1
11	235H	*S. schwetzi*	Female	*Vachellia tortilis*	*Acacia tortilis*	Tree	Fabaceae	100%	100%	MN216557.1
12	241H	*S. schwetzi*	Male	*Solanum lycopersicum*	Tomato plant	Small tree	Solanaceae	100%	98.57%	KX086703.1
13	243H	*S. schwetzi*	Female	*Cenchrus purpureus*	Elephant grass	Grass	Poaceae	100%	100%	MK188842.1
14	254H	*S. schwetzi*	Male	*Vachellia tortilis*	*Acacia tortilis*	Tree	Fabaceae	100%	100%	MN216557.1
15	255H	*S. schwetzi*	Female	*Vachellia tortilis*	*Acacia tortilis*	Tree	Fabaceae	100%	98.81%	MN216557.1
16	257H	*S. antennata*	Female	*Vachellia tortilis*	*Acacia tortilis*	Tree	Fabaceae	100%	100%	MN216557.1
17	264H	*S. africana africana*	Male	*Entada africana*	Tawatsa	Small tree	Fabaceae	100%	96.09%	KX119334.1
18	265H	*S. schwetzi*	Male	*Prosopis juliflora*	Mesquite	Shrub or small tree	Fabaceae	100%	99.81%	MH168722.1
19	283H	*S. squamipleuris*	Female	*Senegalia laeta*	*Acacia laeta*	Shrub or small tree	Fabaceae	100%	99.29%	MN216705.1
20	B32	*P. duboscqi*	Male	*Vachellia tortilis*	*Acacia tortilis*	Tree	Fabaceae	100%	99.82%	MN216557.1
21	D41	*S. schwetzi*	Male	*Laurus nobilis*	Bay laurel	Tree or large shrub	Lauraceae	100%	100%	KX162968.1
22	D51	*S. schwetzi*	Male	*Vachellia tortilis*	*Acacia tortilis*	Tree	Fabaceae	100%	99.82%	MN216557.1
23	E2	*S. schwetzi*	Female	*Eleusine indica*	Indian goosegrass	Small annual grass	Poaceae	100%	95.51%	KX015744.1
24	E3	*S. schwetzi*	Female	*Vachellia tortilis*	*Acacia tortilis*	Tree	Fabaceae	100%	99.82%	MN216557.1

25	E7	*S. schwetzi*	Female	*Senegalia laeta*	*Acacia laeta*	Shrub or small tree	Fabaceae	100%	100%	MN216705.1
26	E31	*S. schwetzi*	Male	*Faidherbia albida*	White acacia	Tree	Fabaceae	100%	96.69%	KX119293.1
27	E33	*S. schwetzi*	Female	*Sporobolus vaginiflorus*	Poverty grass	Grass	Poaceae	100%	94.99%	KJ841600.1
28	E34	*S. schwetzi*	Male	*Musa rosea*	Wild banana	Tree	Musaceae	100%	100%	MG437766.1
29	E38	*P. martini*	Male	*Vachellia tortilis*	*Acacia tortilis*	Tree	Fabaceae	100%	99.82%	MN216557.1
30	E39	*S. schwetzi*	Female	*Chloris virgata*	Feather finger grass	Grass	Poaceae	100%	99.82%	MF998384.1
31	E40	*S. schwetzi*	Male	*Prosopis juliflora*	Mesquite	Perennial shrub or tree	Fabaceae	100%	99.11%	KX015719.1
32	E42	*P. martini*	Female	*Gynerium sagittatum*	Uva Grass	Grass	Poaceae	100%	97.33%	KY549441.1
33	E43	*S. antennata*	Female	*Vachellia tortilis*	*Acacia tortilis*	Tree	Fabaceae	99%	99.80%	MN216557.1
34	E44	*S. schwetzi*	Female	*Senegalia laeta*	*Acacia laeta*	Shrub or small tree	Fabaceae	100%	98.93%	MN216705.1
35	E46	*S. schwetzi*	Female	*Vachellia tortilis*	*Acacia tortilis*	Tree	Fabaceae	100%	99.11%	MN216557.1
36	E48	*S. schwetzi*	Female	*Vachellia tortilis*	*Acacia tortilis*	Tree	Fabaceae	100%	99.02%	MN216557.1
37	E52	*S. schwetzi*	Male	*Prosopis juliflora*	Mesquite	Shrub or small tree	Fabaceae	100%	99.15%	KX015719.1
38	E53	*S. schwetzi*	Female	*Vachellia nilotica*	Gum arabic tree	Tree	Fabaceae	100%	98.04%	LC546141.1
39	M18	*P. martini*	Male	*Vachellia tortilis*	*Acacia tortilis*	Tree	Fabaceae	100%	99.64%	MN216557.1
40	T31	*P. martini*	Male	*Vachellia nilotica*	Gum arabic tree	Tree	Fabaceae	100%	98.75%	LC546141.1
41	P1	-	Plant	*Vachellia tortilis*	*Acacia tortilis*	Tree	Fabaceae	100%	100%	MN216557.1
42	P2	-	Plant	*Senegalia laeta*	*Acacia laeta*	Tree	Fabaceae	100%	100%	MN216705.1
43	P3	-	Plant	*Prosopis juliflora*	Mesquite	Tree	Fabaceae	100%	100%	KX015719.1
44	P4	-	Plant	*Vachellia tortilis*	*Acacia tortilis*	Tree	Fabaceae	100%	100%	MN216557.1
45	P5	-	Plant	*Vachellia tortilis*	*Acacia tortilis*	Tree	Fabaceae	100%	100%	MN216557.1
46	P6	-	Plant	*Vachellia tortilis*	*Acacia tortilis*	Tree	Fabaceae	100%	100%	MN216557.1
47	3_P	-	Plant	*Vachellia sp*	*Acacia* sp	Tree	Fabaceae	100%	100%	MN216486.1

N/A= not applicable

Fig 2. Heatmap depicting the host plant families identified for sand fly species from Rabai village, Barongo County, Kenya.

Correlation in sugars between sand flies and identified plants

Nutritional profiling detected both fructose and sucrose as important sugars present in the leaves of the four identified sand fly host plants (*V. tortilis*, *S. laeta*, *V. nilotica*, *P. juliflora*) (Table 4), although only the former sugar type was present in the gut of sand flies (Table 5; Fig 3). The fructose peaks (5.02 min) were present in the plants in the same ratio as in the sand fly and sucrose was detected at 6.23 min (Fig 3).

Interestingly, sucrose was absent in the leaves of the two *acacia* species *S. senegal* and *V. elatior* which were not among the plants that DNA was detected in sand flies. Fructose content in the leaves significantly varied among the plants examined (F=5.899, df=5, *P*=0.01). Fructose content was highest in *S. senegal* (3-fold) and *P. juliflora* (2-fold) and lowest in *V. tortilis* (0.3-fold). Fructose content was significantly higher for *S. senegal* and *P. juliflora* compared to *V. tortilis*, and between *S. senegal* and *S. laeta* (Table 4). The sucrose content in *P. juliflora* and *V. nilotica* was not significantly different from the other four plants (F=0.61, df=5, *P*=0.63) (Table 4). Fructose was detected in both females and males and the amounts were not significantly different between the female sand fly species (Table 5).

Table 4. Type and amount (ng/mg of sample) of sugars identified in the leaves of selected host plant species in the Fabaceae family

Plant species	Fructose (mean±se)	Sucrose (mean±se)
Vachellia tortilis	7.7 ± 0.9 a	1.4 ± 0.8 ns
Senegalia laeta	10.2 ± 3.1 ab	5.4 ± 3.1 ns
Vachellia nilotica	17.3 ± 2.8 abc	6.5 ± 2.8 ns
Prosopis juliflura	20.5 ± 2.1 bc	9.4 ± 5.4 ns
Vachellia elatior	17.7 ± 3.5 abc	not detected
Senegalia senegal	23.5 ± 1.6 c	not detected

se, standard error; values followed by same letter within a column are not significant at 95% level of significance; ns, not significant

Table 5. Type and amount (ng/mg of sample) of sugars identified in the gut of field collected sand flies

	Fructose content (mean±se)	
**Species**	**Male**	**Female**
P. martini	-	1.8 ± 0.3 ns
S. schwetzi	3.0 ± 0.9	2.5 ± 0.5 ns
S. clydei	3.4 ± 0.9	2.5 ± 0.6 ns
S. antennata	1.4 ± 0.6	1.7 ± 0.3 ns

ns, not significant; se, standard error; -, no data.

Fig 3. HPLC profile of the sugars detected in (a) plant and (b) gut of 50 sand flies. The peaks show names of sugars.

Sand fly host plants vary in their volatile profiles

Analysis of volatiles collected from four of the identified sand fly host plants (*V. tortilis*, *S. laeta*, *V. nilotica*, *P. juliflora*) by GC-MS, detected a total of 26 VOCs during the night and 23 during the day collection. Of these volatiles, 9 were commonly detected in all the plants: benzaldehyde, *β*-pinene, 1-octen-3-ol, benzyl alcohol, (*E*)-*β*-ocimene, acetophenone, *p*-cresol, nonanal and *α*- cedrene (Table 6). The volatiles generally belonged to 7 functional groups: aldehyde, alcohol, benzenoid, ketone, monoterpene and sesquiterpene (Table 6). For the night emissions, 23 VOCs were detected in *P. juliflora*, 19 in *V. tortilis*, 21 in *S. laeta* and 20 in *V. nilotica.* For the day emissions, 18 VOCs were detected in *P. juliflora*, 20, 19 and 16 in *V. tortilis*, *S. laeta* and *V. nilotica*, respectively (Table 6). Overall, the volatile composition of the examined plant hosts did not differ significantly between day and night profiles (one-way ANOSIM based on Bray–Curtis dissimilarity for day ($R = 0.07$, $p = 0.27$) and the night volatile profiles ($R = 0.03$, $p = 0.34$). Clustering of the volatile profiles of the different plants by non-metric multidimensional scaling plot, showed that benzyl alcohol contributed more to the dissimilarity (20.9%) between the different host plant odors followed by (*Z*)-linalool oxide (furanoid) (18.8%), (*E*)-*β*-ocimene (8%), *p*-cymene (6.4%), and *m*-cresol (5%) in the day collection (Fig 4C). The order of contribution of the VOCs to the dissimilarity trends between the plants for the night volatiles were *m*-cresol (16.2%), benzyl alcohol (15.6%), *p*-cresol (13.0), (*E*)-*β*-ocimene (7.4%), and (*Z*)-linalool oxide (furanoid) (7.1%) (Fig 4D).

Fig 4. Non-metric multidimensional scaling plot (NMDS) clustering of the volatile organic compounds during the (A) day and (B) night collection. Histogram depicting the contribution of the five most important volatiles to the differentiation of all the plants types during (C) the day collection and (D) the night collection, based on Analysis of similarities (ANOSIM).

Table 6. Summary of identified volatile organic compounds (VOCs) from four host plants of phelobtomine sand flies in their natural habitats in Rabai village, Baringo County, Kenya.

		Night collection				Day collection						
**Retention time (min)**	**Compound name**	***Prosopis juliflora***	***Vachellia tortilis***	***Senegalia laeta***	***Vachellia nilotica***	***Prosopis juliflora***	***Vachellia tortilis***	***Senegalia laeta***	***Vachellia nilotica***	**Functional group**	**Ri[a]**	**RI[b]L**
6.52	Hexanal	+	+	+	+	-	+	+	-	Aldehyde	805	801
9.14	Heptanal	-	+	+	+	+	+	+	+	Aldehyde	905	907
9.82	*α*-Pinene	+	+	-	-	-	-	-	-	Monoterpene	960	932
10.40	Benzaldehyde	+	+	+	+	+	+	+	+	Benzenoid	1007	965
10.67	Sabinene	+	-	-	-	+	-	-	-	Monoterpene	1029	1017
10.73	*β*-Pinene	+	+	+	+	+	+	+	+	Monoterpene	1033	1008
10.81	1-Octen-3-ol	+	+	+	+	+	+	+	+	Alcohol	1040	1456
10.97	6-methyl-5-hepten-2-one	+	+	+	+	-	+	+	+	Ketone	1053	987
11.27	Octanal	+	+	+	+	-	+	+	+	Aldehyde	1006	1009
11.91	Benzyl alcohol	+	+	+	+	+	+	+	+	Benzenoid	1061	1031
12.02	Octanol	+	-	-	-	-	+	-	-	Alcohol	1013	1561
12.11	(*E*)-*β*-Ocimene	+	+	+	+	+	+	+	+	Monoterpene	1021	1044
12.45	Acetophenone	+	+	+	+	+	+	+	+	Benzenoid	1051	1076
12.57	*m*-Cresol	+	+	+	-	+	+	-	-	Benzenoid	1062	1098
12.64	*p*-Cresol	+	+	+	+	+	+	+	+	Benzenoid	1068	1077
12.85	(*Z*)-linalool oxide (furanoid)	+	-	+	+	+	+	+	+	Monoterpene	1087	1068
13.08	Nonanal	+	+	+	+	+	+	+	+	Aldehyde	1107	1087
13.36	*p*-Cymene	+	-	+	+	+	+	+	-	Monoterpene	1132	1014
14.15	(*Z*)-Linalool oxide (pyranoid)	+	+	+	-	-	-	-	-	Monoterpene	1160	1095
14.57	Methyl salicylate	+	+	-	+	+	+	+	+	Benzenoid	1198	1190
14.68	Decanal	+	+	+	+	+	+	+	+	Aldehyde	1209	1203
16.03	Indole	-	-	-	+	-	-	+	+	Benzenoid	1299	1298
17.66	Longifolene	+	+	+	+	+	+	+	-	Sesquiterpene	1425	1406

17.74	*α*- Cedrene	+	+	+	+	+	+	+	+	Sesquiterpene	1433	1413
19.89	Phytol	-	-	+	-	-	-	-	-	Monoterpene	1603	2223
20.12	epi-Cedrol	+	-	+	+	+	-	-	-	Sesquiterpene	1625	1611

(RT) = retention times

RI[a] = Retention index relative to C8-C23 n- alkanes of a HP-5 MS column

RI[b]L = Retention index obtained from literature: [30–32]

(+) = compound present and (-) = compound absent

Discussion

Here, we report the feeding patterns of wild-caught sand flies from a leishmaniasis endemic area in a dry ecology of Kenya. There was no sex bias in the feeding rates indicating the importance of this trait in the biology of both males and females as previously observed [6,11]. Analysis of derived rbcL sequences implicated acacias as predominant plants readily foraged on by both sand fly sexes of diverse *Phlebotomus* and *Sergentomyia* species. Preference for feeding on plants in the Fabaceae family to which acacia plants belong, has been documented in sand flies [15], and the malaria vector *Anopheles gambiae* [35]. However, both extent and possibly choice may be influenced by season as demonstrated in other mosquito species [36].

The vegetation cover in the study area has little undergrowth mainly with *Acacia* plants and weeds. Whether proclivity on acacia is related to their abundance in the area is unclear. We found the presence of other plants in the area during the trapping period that could potentially serve as feeding sources based on succulent stems or leaves. These included *Opuntia stricta*, *Kalanchoe lanceolate*, *Euphorbia gossypina*, *Agave americana*, *Indigofera volkensii*, *Aloe secundiflora*, *Maerua subcordata*, *Caralluma russeliana* and *Balanites aegyptiaca*. Moreover, the invasive plant *P. juliflora* is rapidly spreading in the area and displacing the indigenous species such as acacias. Yet, we found only three specimens that had fed on this plant based on analyzed rbcL sequences. Other adaptations than abundance may explain the seeming strong association between acacias and diverse sand fly species at the study site during the study period. Acacia plants must possess unique adaptive qualities to explain the observed utilization not only by sand flies as observed in our study but also mosquito species [35]. Previously, high abundance of *P. orientalis* in *Acacia seyal* vegetation were attributed to factors such as the tree density and soil type and other microclimatic factors either operating independently or in combination to provide a suitable habitat for the vector (reviewed in [17], as well as nutrition .

Like mosquitoes, sand fly herbivory has an underlying nutrient benefit but also a semiochemical basis. Sugar feeding is of prime relevance in the transmission of *Leishmania* parasites in sand flies (Killick- Kendrick and Killick- Kendrick 1987). We compared the sugar content present in the guts of groups of males and females and that of six selected plants in the family Fabaceae (*V. tortilis*, *S. laeta*, *V. nilotica* and *P. juliflora*, *S. senegal* and *V. elatior*). Of 8 sugars tested by HPLC, only fructose was commonly detected in the sand flies as well as leaves of these plants, indicating that this sugar is a common constituent of their diet. It is unclear if sucrose presence is a discriminating factor for feeding by sand flies given that it was

not detected in acacia species absent in their plant meals. Sucrose and trehalose have previously been reported as an important constituent of sand fly diet [38]. Maltose was also absent from their gut. The sugars detected may not represent the full profile in the insect [38] given the range of sugars tested. The detection of plant DNA confirms plant tissue feeding which sand flies generally imbibe by direct piercing and sucking [39,40]. Our analysis showed that, vegetative tissue may serve as a source of sugar, given the similarity in the detection of fructose between tissue extract and sand fly gut contents. Thus, specific sugar profiles in sand flies likely reflects those of the sources they commonly imbibe. The findings indicate that sand flies readily feed on the plant tissue to obtain these nutrients. *Vachellia tortillis* was the predominant acacia in which plant DNA was detected in sand flies. However, this plant had the lowest content of fructose and sucrose (Table 4) although worth noting that these profiles may be dynamic. Manda et al [41], found that the preferred plants for the malaria vector *An. gambiae*, had the most sugars; this is in contrast to our findings given the sampling period. The variation in sugar types and amounts between plants suggest varied nutrient benefits and could potentially have phenotypic impact on sand fly bionomics including longevity and egg laying as observed for mosquitoes [42].

There are indications that besides sugars, acacias are generally rich in tannins which have been found to be particularly high in their leaves and fruits and occurring in varying amounts in different species [43]. Influence of tannins on the growth and fecundity of some insects and microbe interaction has previously been reported [44]. Further assessment of this specific sand fly-acacia interaction in relation to pathogen transmission could be a useful area for further research.

Despite knowledge of sand fly plant association, there have been minimal studies conducted to examine the semiochemical basis. Evidence from mosquito vectors have demonstrated the major roles played by olfactory cues emitted by suitable host plants that attract vectors for feeding [10,45–47]. Examining the volatile profiles of selected identified plants in the Fabaceae family via GC-MS showed differences in VOC compositions between the plant species collected during the night- and day-time (Fig 4). Of the identified discriminating volatile compounds (benzyl alcohol, (*Z*)-linalool oxide (furanoid), (*E*)-β-ocimene, *p*-cymene, and *p*/*m*-cresol), (*E*)-*β*-ocimene has been implicated as a signature cues for plant host location in diverse mosquito species [13]. Linalool oxide, a typical plant volatile, is known as an attractant for the mosquitoes *Aedes aegypti* [3,48] and *Anopheles gambiae* sl [49,50]. The communicative function of these compounds in sand flies is yet to be established. Other compounds present in the volatile emissions of these plants including the alcohols 1-

octen-3-ol, octanol and ketone 6-methyl-5-hepten-2-one have been found to elicit behavioral activity in sand flies either in the laboratory or field setting [51–53]. However, these compounds present as complex mixtures in differing amounts and ratios could interact to influence sand fly attraction to a given plant species. Electrophysiological and behaviroal assays should be conducted on these VOCs to identify the attractants that can be used in monitoring sand fly populations.

Widely distributed in East Africa, sand flies are important vectors of diseases such as leishmaniasis where increased frequency of outbreaks has been reported in recent times [1]. This is a trend reflected in part by inadequacies of current chemotherapeutic approaches to curb the disease spread. The profile of sand fly vectored pathogens includes phleboviruses as demonstrated elsewhere and in Kenya recently [54–56] and without vaccines. The present findings provide clues towards the development of new approaches including use of the attractants in bait technologies such as Attractive Toxic Sugar Baits (ATSBs) for sand fly control.

While the data generated suggest selective sand fly feeding, the success rate of plant DNA detection including sequencing from fructose positive sandflies was low (<6%). Similarly, low rates using the same rbcL gene marker were reported by Abbasi et al [16] and Lima et al [15] analyzing wild-caught sand flies. The reasons for this observation could be ascribed to enzymatic degradation of DNA in the gut of sand flies or sugar feeding from other sources including secretions from aphids and coccids, that lack DNA [15,39,57]. More sensitive high-throughput methodologies such as next generation sequencing [14], could improve the profile of plant sequences.

In summary, our study demonstrates that Afrotropical phlebotomine sand flies including the leishmaniasis vectors *P. duboscqi* and *P. martini* of both sexes predominantly feed on plants in the Fabaceae family in a dry ecology, of which *Acacias* are predominantly represented. Only fructose was commonly detected in the gut of sand flies and plant vegetative parts, likely indicating that the specific sugar profile in sand flies reflects those of the plant sources they commonly feed on (Fabaceae). Future studies on the role VOCs play in plant feeding should greatly enhance our understanding of the olfactory system of sand flies and their use for management purposes.

Acknowledgments

We are grateful for the technical support of Caroline Kungu, Jackson M. Muema, Dr. Joseph Gichuhi and Mr. James Kabii. We would also like to acknowledge the help and support received from Dr. Fathiya M. Khamis, Souleymane Diallo and Simon Kiplimo.

References

1. WHO. Leishmaniasis.World Health Organization. 2020. Available: https://www.who.int/news-room/fact-sheets/detail/leishmaniasis
2. Munyua P, Bitek A, Osoro E, Pieracci EG, Muema J, Mwatondo A, Kungu M, Nanyingi M, Gharpure R, Njenga K, Thumbi SM. Prioritization of zoonotic diseases in Kenya, 2015. PLoS One. 2016;11(8): e0161576. doi:10.1371/journal.pone.0161576
3. Omondi WP, Owino EA, Odongo D, Mwangangi JM, Torto B, Tchouassi DP. Differential response to plant- and human-derived odorants in field surveillance of the dengue vector, *Aedes aegypti*. Acta Trop. 2019;200: 105163. doi:10.1016/j.actatropica.2019.105163
4. Kanyina EW. Characterization of visceral leishmaniasis outbreak, Marsabit County, Kenya, 2014. BMC Public Health. 2020;20: 446. doi:10.1186/s12889-020-08532-9
5. Warburg A, Faiman R. Research priorities for the control of phlebotomine sand flies. J Vector Ecol. 2011;36: 10–16. doi:10.1111/j.1948-7134.2011.00107.x
6. Schlein Y, Jacobson RL. Mortality of *leishmania* in *phlebotomus* of the sand flies. Am J Trop Med Hyg. 1994;50: 20–27.
7. Schlein Y, Müller GC. Experimental control of *Phlebotomus papatasi* by spraying attractive toxic sugar bait (ATSB) on vegetation. Trans R Soc Trop Med Hyg. 2010;104: 766–771. doi:10.1016/j.trstmh.2010.08.014
8. Müller GC, Schlein Y. Different methods of using attractive sugar baits (ATSB) for the control of *Phlebotomus papatasi*. J Vector Ecol. 2011;36: S64–S70. doi:10.1111/j.1948-7134.2011.00113.x
9. Qualls WA, Müller GC, Revay EE, Allan SA, Arheart KL, Beier JC, et al. Evaluation of attractive toxic sugar bait (ATSB)-Barrier for control of vector and nuisance mosquitoes and its effect on non-target organisms in sub-tropical environments in Florida. Acta Trop. 2014;131: 104–110. doi:10.1016/j.actatropica.2013.12.004
10. Torto B. Innovative approaches to exploit host plant metabolites in malaria control. Pest Manag Sci. 2019;75: 2341–2345. doi:10.1002/ps.5460

11. Junnila A, Müller GC, Schlein Y. Attraction of *Phlebotomus papatasi* to common fruit in the field. J Vector Ecol. 2011;36: S206–S211. doi:10.1111/j.1948-7134.2011.00132.x
12. Beier JC, Müller GC, Gu W, Arheart KL, Schlein Y. Attractive toxic sugar bait (ATSB) methods decimate populations of Anopheles malaria vectors in arid environments regardless of the local availability of favoured sugar-source blossoms. Malar J. 2012;11: 31. doi:10.1186/1475-2875-11-31
13. Nyasembe VO, Tchouassi DP, Pirk CWW, Sole CL, Torto B. Host plant forensics and olfactory-based detection in Afro-tropical mosquito disease vectors. PLoS Negl Trop Dis. 2018;12: e0006185. doi:10.1371/journal.pntd.0006185
14. Abbasi I, Nasereddin A, Warburg A. Development of a next generation DNA sequencing-based multi detection assay for detecting and identifying *Leishmania* parasites, blood sources, plant meals and intestinal microbiome in phlebotomine sand flies. Acta Trop. 2019;199: 105101. doi:10.1016/j.actatropica.2019.105101
15. Lima LHG de M, Mesquita MR, Skrip L, de Souza Freitas MT, Silva VC, Kirstein OD, Abassi I, Warburg A, Balbino VD, Costa CH. DNA barcode for the identification of the sand fly *Lutzomyia longipalpis* plant feeding preferences in a tropical urban environment. Sci Rep. 2016;6: 29742. doi:10.1038/srep29742
16. Abbasi I, Trancoso Lopo de Queiroz A, Kirstein OD, Nasereddin A, Horwitz BZ, Hailu A, Salah I, Mota TF, Fraga DB, Veras PS, Poche D. Plant-feeding phlebotomine sand flies, vectors of leishmaniasis, prefer *Cannabis sativa*. Proc Natl Acad Sci. 2018;115: 11790–11795. doi:10.1073/pnas.1810435115
17. Elnaiem DEA. Ecology and control of the sand fly vectors of Leishmania donovani in East Africa, with special emphasis on *Phlebotomus orientalis.* J Vector Ecol. 2011;36: 23–31. doi:10.1111/j.1948-7134.2011.00109.x
18. Anjili CO, Ngumbi PM, Kaburi JC, Irungu LW. The phlebotomine sandfly fauna (Diptera: Psychodidae) of Kenya. J Vector Borne Dis. 2011;48: 183–189.
19. Tchouassi DP, Sang R, Sole CL, Bastos AD, Cohnstaedt LW, Torto B. Trapping of Rift Valley Fever (RVF) vectors using Light Emitting Diode (LED) CDC traps in two arboviral disease hot spots in Kenya. Parasite Vectors. 2012;5: 94. doi:10.1186/1756-3305-5-94
20. Kirk K, Lewis DJ. The phlebotominae of the Ethiopian region. Trans R Entomol Soc London. 1951;102: 383–510. doi:10.1111/j.1365-2311.1951.tb00759.x
21. Abonnenc E, Minter D M. Bilingual keys for the identification of the sandflies of the ethiopian region. Cah ORSTOM, (Entomologie medicale). 1965;5: 1–63.

22. Van Handel E. The detection of nectar in mosquitoes. Mosquito News. 1972.
23. Matheson CD, Muller GC, Junnila A, Vernon K, Hausmann A, Miller MA, Greenblatt C, Schlein Y. A PCR method for detection of plant meals from the guts of insects. Org Divers Evol. 2008;7: 294–303. doi:https://doi.org/10.1016/j.ode.2006.09.002
24. Levin RA, Wagner WL, Hoch PC, Nepokroeff M, Pires JC, Zimmer EA, Sytsma KJ. Family-level relationships of Onagraceae based on chloroplast rbcL and ndhF data. Am J Bot. 2003;90: 107–115. doi:10.3732/ajb.90.1.107
25. Kress WJ, Erickson DL. A Two-Locus global DNA barcode for land plants: the coding rbcL gene complements the non-coding trnH-psbA spacer region. PLoS One. 2007;2: e508. doi:10.1371/journal.pone.0000508
26. Kumar S, Stecher G and TK. MEGA7: Molecular evolutionary genetics analysis version 7.0 for bigger datasets. Mol Biol Evol. 2016;33: 1870–1874.
27. Agha SB, Tchouassi DP, Turell MJ, Bastos ADS, Sang R. Entomological assessment of dengue virus transmission risk in three urban areas of Kenya. PLoS Negl Trop Dis. 2019;13: e0007686. doi:10.1371/journal.pntd.0007686
28. Mokaya HO, Njeru LK, Lattorff HMG. African honeybee royal jelly: Phytochemical contents, free radical scavenging activity, and physicochemical properties. Food Biosci. 2020;37: 100733. doi:10.1016/j.fbio.2020.100733
29. van Den Dool H, Dec. Kratz P. A generalization of the retention index system including linear temperature programmed gas—liquid partition chromatography. J Chromatogr. 1963;11: 463–471. doi:10.1016/S0021-9673(01)80947-X
30. Karioti A, Hadjipavlou-Litina D, Mensah MLK, Fleischer TC, Skaltsa H. Composition and antioxidant activity of the essential oils of *Xylopia aethiopica* (Dun) A. Rich. (Annonaceae) leaves, stem bark, root bark, and fresh and dried fruits, growing in Ghana. J Agric Food Chem. 2004;52: 8094–8098. doi:10.1021/jf040150j
31. Babushok VI, Linstrom PJ, Zenkevich IG. Retention indices for frequently reported compounds of plant essential oils. J Phys Chem Ref Data. 2011;40: 043101:1–47. doi:10.1063/1.3653552
32. Njuguna PK, Murungi LK, Fombong A, Teal PEA, Beck JJ, Torto B. Cucumber and tomato volatiles: influence on attraction in the melon fly *Zeugodacus cucurbitate* (Diptera: Tephritidae). J Agric Food Chem. 2018;66: 8504–8513. doi:10.1021/acs.jafc.8b03452
33. Team RC. R: A Language and environment for statistical computing. In: R foundation for statistical computing.Vienna, Austria. 2020. Available: https://www.r-project.org/

34. Hammer Ø, Harper D A, Ryan PD. Past: paleontological statistics software package for education and data analysis. Palaeontol Electron. 2001;4: 9.

35. Müller GC, Beier JC, Traore SF, Toure MB, Traore MM, Bah S, et al. Field experiments of *Anopheles gambiae* attraction to local fruits/seedpods and flowering plants in Mali to optimize strategies for malaria vector control in Africa using attractive toxic sugar bait methods. Malar J. 2010;9: 1–11. doi:10.1186/1475-2875-9-262

36. Olson MF, Garcia-Luna S, Juarez JG, Martin E, Harrington LC, Eubanks MD, Badillo-Vargas IE, Hamer GL. Sugar Feeding Patterns for *Aedes aegypti* and *Culex quinquefasciatus* (Diptera: Culicidae) Mosquitoes in South Texas. Scott T, editor. J Med Entomol. 2020;57: 1111–1119. doi:10.1093/jme/tjaa005

37. Killick- kendrick R, Killick- kendrick M. Honeydew of aphids as a source of sugar for *Phlebotomus ariasi*. Med Vet Entomol. 1987;1: 297–302. doi:10.1111/j.1365-2915.1987.tb00358.x

38. Hamilton JGC, El Naiem DA. Sugars in the gut of the sandfly *Phlebotomus orientalis* from Dinder National Park, Eastern Sudan. Med Vet Entomol. 2000;14: 64–70. doi:10.1046/j.1365-2915.2000.00208.x

39. Junnila A, Müller GC, Schlein Y. Identification of plant tissues from the gut of *Phlebotomus papatasi* by DNA analysis. Acta Trop. 2011;117: 14–18. doi:https://doi.org/10.1016/j.actatropica.2010.08.019

40. Schlein Y, Warburg A, Yuval B. On the system by which sandflies maintain a sterile gut. Int J Trop Insect Sci. 1986;7: 231–234. doi:10.1017/S1742758400009000

41. Manda H, Gouagna LC, Nyandat E, Kabiru EW, Jackson RR, Foster WA, Githure JI, Beier JC, Hassanali A. Discriminative feeding behaviour of *Anopheles gambiae* s.s. on endemic plants in western Kenya. Med Vet Entomol. 2007;21: 103–111. doi:10.1111/j.1365-2915.2007.00672.x

42. Manda H, Gouagna LC, Foster WA, Jackson RR, Beier JC, Githure JI, Hassanali A. Effect of discriminative plant-sugar feeding on the survival and fecundity of *Anopheles gambiae*. Malar J. 2007;6: 113. doi:10.1186/1475-2875-6-113

43. Elgailani IEH, Ishak CY. Determination of tannins of three common *Acacia* species of Sudan. Adv Chem. 2014;2014: 1–5. doi:10.1155/2014/192708

44. Schultz JC. Tannin-Insect Interactions. Chemistry and significance of condensed Tannins. Boston, MA: Springer US. 1989;417–433. doi:10.1007/978-1-4684-7511-1_26

45. Nyasembe VO, Teal PEA, Mukabana WR, Tumlinson JH, Torto B. Behavioural

response of the malaria vector *Anopheles gambiae* to host plant volatiles and synthetic blends. Parasit Vectors. 2012;5: 234. doi:10.1186/1756-3305-5-234

46. Nyasembe VO, Torto B. Volatile phytochemicals as mosquito semiochemicals. Phytochem Lett. 2014;8: 196–201. doi:10.1016/j.phytol.2013.10.003

47. Lahondère C, Vinauger C, Okubo RP, Wolff GH, Chan JK, Akbari OS, Riffell JA. The olfactory basis of orchid pollination by mosquitoes. Proc Natl Acad Sci. 2020;117: 708–716. doi:10.1073/pnas.1910589117

48. Nyasembe VO, Tchouassi DP, Mbogo CM, Sole CL, Pirk C, Torto B. Linalool oxide: generalist plant based lure for mosquito disease vectors. Parasit Vectors. 2015;8: 581. doi:10.1186/s13071-015-1184-8

49. Nyasembe VO, Tchouassi DP, Kirwa HK, Foster WA, Teal PEA, Borgemeister C, Torto B. Development and assessment of plant-based synthetic odor baits for surveillance and control of malaria vectors. Leal WS, editor. PLoS One. 2014;9: e89818. doi:10.1371/journal.pone.0089818

50. Jacob JW, Tchouassi DP, Lagat ZO, Mathenge EM, Mweresa CK, Torto B. Independent and interactive effect of plant- and mammalian- based odors on the response of the malaria vector, *Anopheles gambiae*. Acta Trop. 2018;185: 98–106. doi:10.1016/j.actatropica.2018.04.027

51. Magalhães-Junior JT, Barrouin-Melo SM, Corrêa AG, Da Rocha Silva FB, Machado VE, Govone JS, Pinto MC. A laboratory evaluation of alcohols as attractants for the sandfly *Lutzomyia longipalpis* (Diptera:Psychodidae). Parasit Vectors. 2014;7: 60. doi:10.1186/1756-3305-7-60

52. Machado V, Corrêa A, Goulart T, Silva F, Ortiz D, Pinto M. Attraction of the sand fly *Nyssomyia neivai* (Diptera: Psychodidae) to chemical compounds in a wind tunnel. Parasit Vectors. 2015;8: 147. doi:10.1186/s13071-015-0748-y

53. Da Silva Tavares D, Salgado VR, Miranda JC, Mesquita PRR, De Medeiros Rodrigues F, Barral-Netto M, de Andrade JB, Barral A. Attraction of phlebotomine sandflies to volatiles from skin odors of individuals residing in an endemic area of tegumentary leishmaniasis. PLoS One. 2018;13(9): e0203989. doi:10.1371/journal.pone.0203989

54. Marklewitz M, Tchouassi DP, Hieke C, Heyde V, Torto B, Sang R, Junglen S. Insights into the evolutionary origin of mediterranean sandfly fever viruses. Spiropoulou CF, editor. mSphere. 2020;5: e00598-20. doi:10.1128/msphere.00598-20

55. Tchouassi DP, Marklewitz M, Chepkorir E, Zirkel F, Agha SB, Tigoi CC, Koskei E, Drosten C, Borgemeister C, Torto B, Junglen S. Sand fly-associated *Phlebovirus* with

evidence of neutralizing antibodies in humans, kenya. Emerg Infect Dis. 2019;25: 681–690. doi:10.3201/eid2504.180750

56. Marklewitz M, Dutari LC, Paraskevopoulou S, Page RA, Loaiza JR, Junglen S. Diverse novel phleboviruses in sandflies from the Panama Canal area, Central Panama. J Gen Virol. 2019;100: 938–949. doi:10.1099/jgv.0.001260

57. Schlein Y, Warburg A. Phytophagy and the feeding cycle of *Phlebotomus papatasi* (Diptera: Psychodidae) under experimental conditions. J Med Entomol. 1986;23: 11–15. doi:10.1093/jmedent/23.1.11

Supporting Information

S1 Fig. Collection of headspace volatiles from sand fly host plants in the field in Rabai village, Marigat sub-county, Kenya

Figure

Click here to access/download;Figure;Fig 1.tif

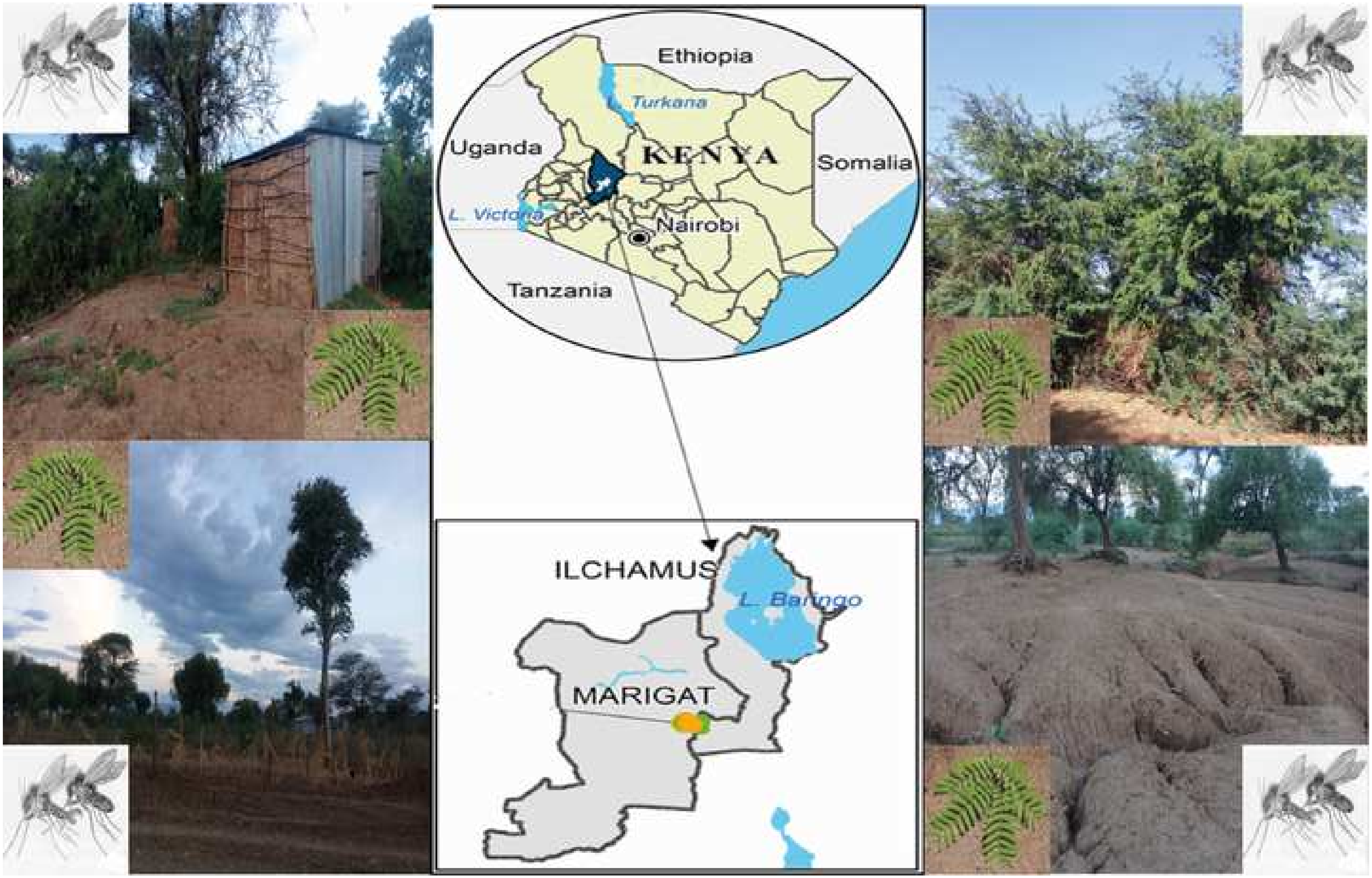

Click here to access/download;Figure;Fig 2.tif

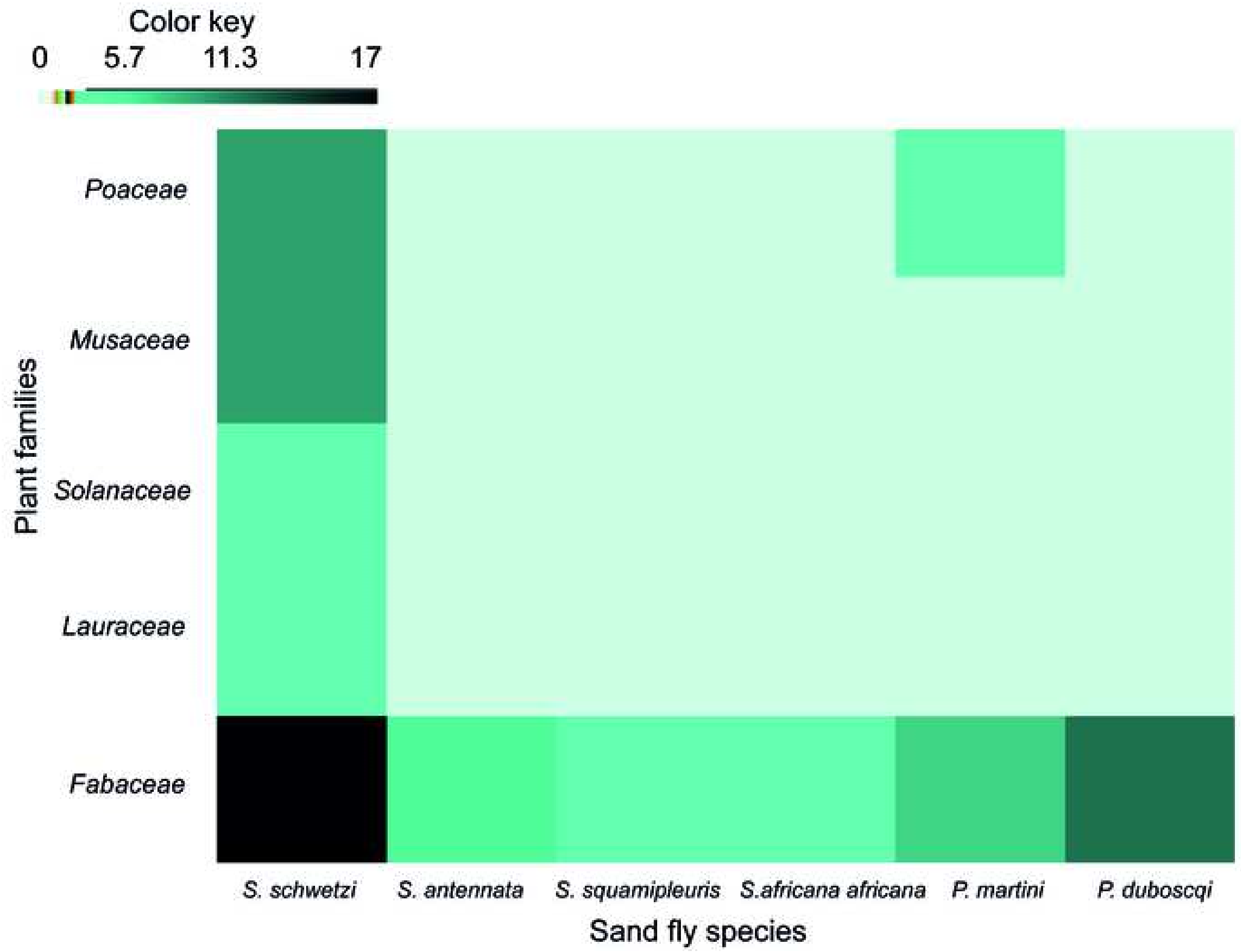

Figure

Click here to access/download;Figure;Fig 3.tif

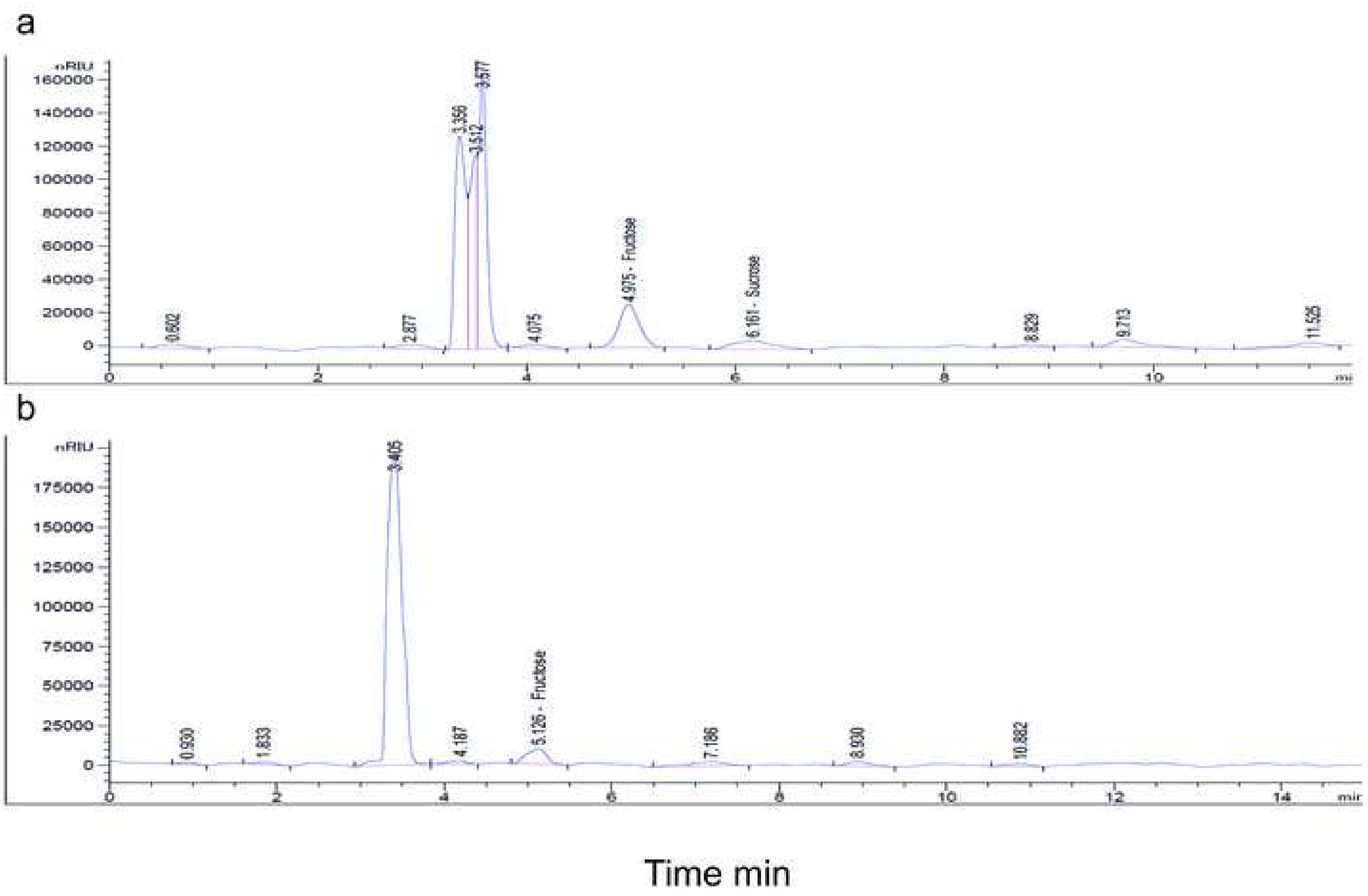

 Click here to access/download;Figure;Fig 4.tif

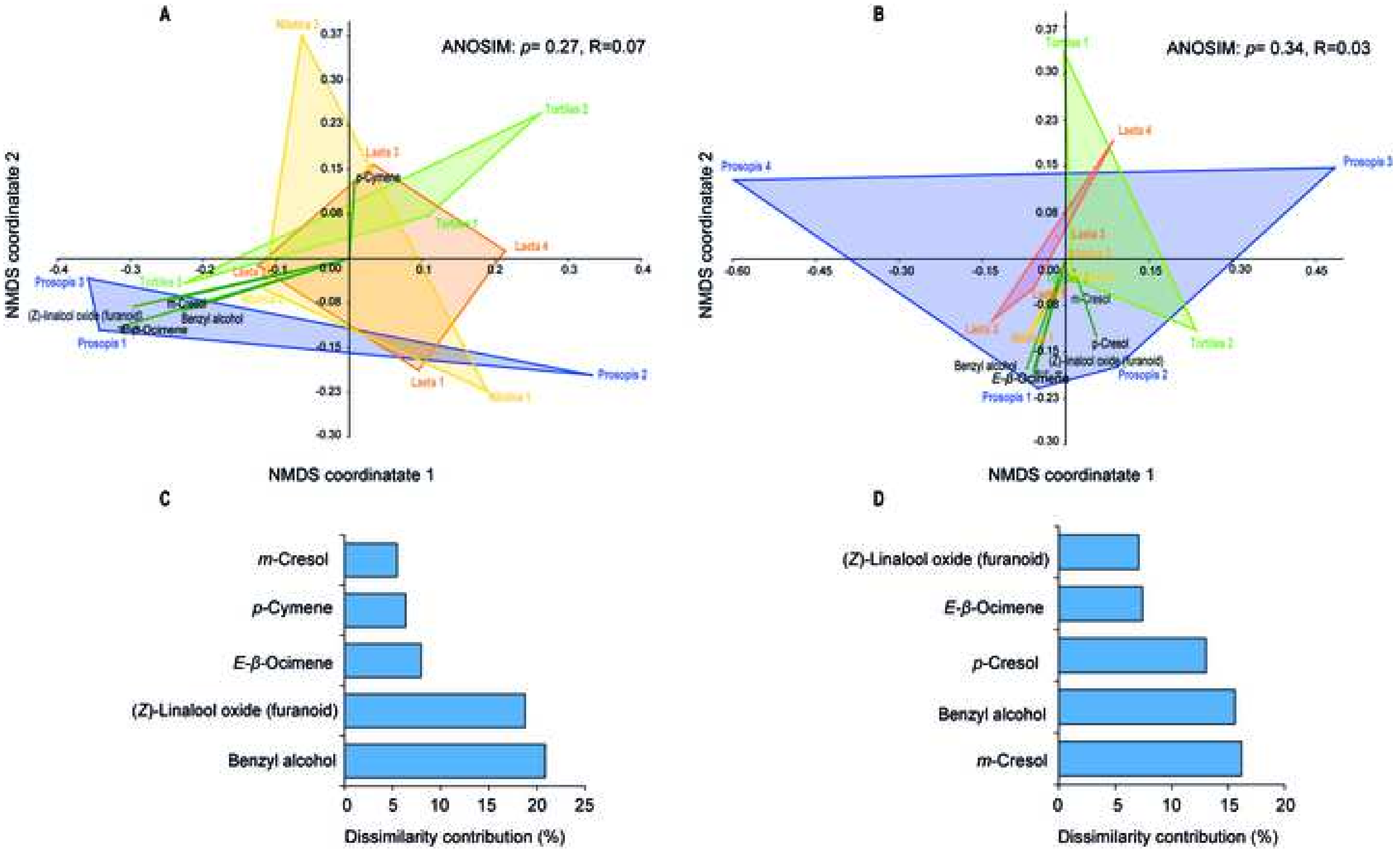

Click here to access/download
Supporting Information
S1 Fig.tif
