# Supplementary material for: Afrotropical sand fly-host plant relationships in a leishmaniasis endemic area, Kenya
Source: PLoS Negl Trop Dis. 2021 Feb 8;15(2):e0009041. doi: 10.1371/journal.pntd.0009041 (PMC7895382; doi:10.1371/journal.pntd.0009041)
Supplement: Attachment — Submitted filename: Response to reviewers comments 14122020F.docx [file pntd.0009041.s005.docx]

14^th^ December 2020.

Editor-in-Chief,

PLoS Neglected Tropical Diseases,

Shaden Kamhawi (PhD).

Dear Prof Kamhawi,

**Re: Manuscript re-submission: PNTD-D-20-01826**

We are pleased to resubmit the manuscript entitled **"**Afrotropical sand fly-host plant interactions in a leishmaniasis endemic area, Kenya" for further consideration for publication in your journal. We have made all the changes you suggested in your letter and addressed the comment of the Reviewers in the notes below. Please note that the reviewers’ comments are shown in bold type and our responses in plain type italics.

Yours sincerely,

David P. Tchouassi

Dear Dr Tchouassi,
 
Thank you very much for submitting your manuscript "Afrotropical sand fly-host plant interactions in a leishmaniasis endemic area, Kenya" for consideration at PLOS Neglected Tropical Diseases. As with all papers reviewed by the journal, your manuscript was reviewed by members of the editorial board and by several independent reviewers. The reviewers appreciated the attention to an important topic. Based on the reviews, we are likely to accept this manuscript for publication, providing that you modify the manuscript according to the review recommendations.
 
Please prepare and submit your revised manuscript within 30 days. If you anticipate any delay, please let us know the expected resubmission date by replying to this email.  

When you are ready to resubmit, please upload the following:

[1] A letter containing a detailed list of your responses to all review comments, and a description of the changes you have made in the manuscript. 
Please note while forming your response, if your article is accepted, you may have the opportunity to make the peer review history publicly available. The record will include editor decision letters (with reviews) and your responses to reviewer comments. If eligible, we will contact you to opt in or out

[2] Two versions of the revised manuscript: one with either highlights or tracked changes denoting where the text has been changed; the other a clean version (uploaded as the manuscript file).

Important additional instructions are given below your reviewer comments.

Thank you again for your submission to our journal. We hope that our editorial process has been constructive so far, and we welcome your feedback at any time. Please don't hesitate to contact us if you have any questions or comments.
 
Sincerely,

Shan Lv, Ph.D.
Deputy Editor
PLOS Neglected Tropical Diseases

Shan Lv
Deputy Editor
PLOS Neglected Tropical Diseases

***********************

Reviewer's Responses to Questions

<b>Key Review Criteria Required for Acceptance?</b></br></br>
As you describe the new analyses required for acceptance, please consider the following:</br></br>

<b>Methods</b></br></br>
-Are the objectives of the study clearly articulated with a clear testable hypothesis stated?</br>
-Is the study design appropriate to address the stated objectives?</br>
-Is the population clearly described and appropriate for the hypothesis being tested?</br>
-Is the sample size sufficient to ensure adequate power to address the hypothesis being tested?</br>
-Were correct statistical analysis used to support conclusions?</br>
-Are there concerns about ethical or regulatory requirements being met?</br></br>

**Reviewer #1: The methods were clearly articulated and included sufficient detail for reproducing the experiments. The implied hypothesis is that sand flies are selective in their acquisition of dietary sugar sources.  Given the outcome that Vachellia tortillis was the preferred sugar source, even though it had the lowest content, it would be good to add something in the study about the relative abundance of the different plants in the study area.**

*Re: We have rewritten this part to read “Whether proclivity on Acacia is related to its biological traits, that is, a tree with a large biomass containing a range of nutrients, which may present it as a more important available and accessible food source than many small bushes or grasses or weeds to sandflies, is unclear. More research is needed to unravel the strong association with Acacia. L439-443.*

**Reviewer #2: -yes
-yes
-a little small but none the less indicative
-a little small but none the less indicative (allowances must be afforded for the inherent difficulties in collecting and procesing large numbers of insects in the middle of the African bush)
- I am not qualified to ascertain statistical data
-none**

*Re: No response needed*

**Reviewer #3: The study is well delineated with the objectives very clear and the methodology appropriated to test the hypothesis. I did not detect problems with the statistical analysis or other techniques carried out. No concern related to ethical or regulatory requirements.**

*Re: No response needed*

<b>Results</b></br></br>
-Does the analysis presented match the analysis plan?</br>
-Are the results clearly and completely presented?</br>
-Are the figures (Tables, Images) of sufficient quality for clarity?</br></br>

**Reviewer #1: The results are clearly presented and mostly easy to understand.  However, in Table 4 under the Fructose column, it would be helpful to define what 'a', 'ab', 'bc' etc mean at the bottom of the table.**

*Re: Based on mean separation, the amount of fructose is compared between the plants and the letters beside each value used to indicate which pairs of treatment (plants) are significantly different from each other. This explanation has been provided in the table viz: ….values followed by same letter within a column are not significantly different at 95% level of significance. Table 3. L 383-384*

**Reviewer #2: - N/A
- yes (some comments in the attached MS)
- In my opinion tables may be condensed and the full tables relegated to supplementary data**

*Re: Table 3 has been revised and now included as a supplementary table (S1Table). Removed from ms*

**Reviewer #3: The results are clearly presented. Only the Fig 4 A and B do not present a good resolution.**

*Re: Fig 4A and B have been revised to improve resolution*

<b>Conclusions</b></br></br>
-Are the conclusions supported by the data presented?</br>
-Are the limitations of analysis clearly described?</br>
-Do the authors discuss how these data can be helpful to advance our understanding of the topic under study?</br>
-Is public health relevance addressed?</br></br>

**Reviewer #1: The conclusions are appropriately supported by the data and analysis.  Public health relevance is clearly acknowledged in the introduction and discussion.  However, this sentence at line numbers 496 - 497 is a bit unclear: "The profile of sand fly vectored pathogens includes phleboviruses as demonstrated elsewhere and in Kenya recently [54–56] and without vaccines." Are you implying here that there are no vaccines for protection against phleboviruses? or that in this part of Kenya, people are not vaccinated?  I think this sentence could be restated for clarity.**

*Re: The sentence has been revised and now reads…The profile of sand fly vectored pathogens includes phleboviruses as demonstrated elsewhere and in Kenya recently [54–56] and there are no vaccines for the diseases they cause. L 509-510*

**Reviewer #2: - yes
- I dont know
- yes
-yes**

*Re: No response needed*

**Reviewer #3: The conclusions are supported by the results and the authors discuss possible bias of the study. The investigation of basic biology of sand flies present relevance for public health, since the control of these insects need different and new approaches.**

*Re: No response needed*

<b>Editorial and Data Presentation Modifications?</b></br><br/>
Use  this section for editorial suggestions as well as relatively minor modifications of existing data that would enhance clarity. If the only modifications needed are minor and/or editorial, you may wish to recommend “Minor Revision” or “Accept”.

**Reviewer #1: A few minor edits:**

**Line 93 - "Male sand flies are exclusively feed on plants." Remove the word "are"**

*Re: Corrected L95*

**Line 120 - add a period at the end of this sentence.**

*Re: Corrected L122*

**Line 134 - consider changing "icipe" to "International Centre of Insect Physiology and Ecology (ICIPE)" and then use ICIPE throughout the rest of the document.**

*Re: Corrected L136-137, 198, 211*

**Line 187 - change "Blanstn" to "****BLASTn"**

*Re: Corrected L191*

**Line 235 – remove the ‘a’ from “to generate a linear calibration curves”**

*Re: Corrected L235*

**Line 291 – you need a period after the word “Aldrich”**

*Re: Corrected L291*

**Line 447 – Use the same referencing convention used in the rest of the paper [##]**

*Re: Corrected L 459*

**Line 460 – “likely reflects” should be “likely reflect”**

*Re: Corrected L 472*

**Line 482 - remove the 'a' from "implicated as a signature cues"**

*Re: Corrected L 494*

**Line 484 - what does the 'sl' mean in "Anopheles gambiae sl"?**

*Re: Corrected to read senso lato (s.l.) signifying a species complex. L 497*

**Line 490 – “behaviroal” should be “behavioral”**

*Re: Corrected L 502*

**Line 497 And without vaccine**

*Re: Corrected L503-504 (there are no vaccines for the diseases they cause)*

**perhaps you should add what 'a', 'ab', 'abc', etc means**

*Re: Based on mean separation, the amount of fructose is compared between the plants and the letters beside each value used to indicate which pairs of treatment (plants) are significantly different from each other. This explanation has been provided in the table viz: values followed by same letter within a column are not significantly different at 95% level of significance. Table 3. L 383-384*

**Reviewer #2: see attached ms**

*Re: Corrected L 69(L) (Leishmania)*

*Re: Corrected L 95 (*Male sand flies feed exclusively on plants*)*

*Re: Corrected L 105 (deleted success)*

*Re: Corrected L 128 (ecological zone)*

*Re: Corrected L 129 (mostly and grazed)*

*Re: Corrected L 130 (add common name Kanniedood)*

*Re: Corrected L 133 (deleted)*

*Re: Corrected L 136 (add (,) (immediately, frozen)*

*Re: Corrected (ICIPE) line 136-137, 198, 211*

*Re: Corrected L 145 (pharynx, cibarium)*

*Re: Corrected L 149 (E) (Evidence of recent plant feeding in the field sand flies)*

*Re: Corrected L 149 (deleted collection) (Evidence of recent plant feeding in the field sand flies)*

*Re: Corrected L 156-157 (blue was deemed)*

*Re: Corrected L 212 (in the) instead (under). They were separately air-dried in the shade at room temperature*

*Re: Corrected L250-251 (add species and leaf) (for each plant species using a different plant leaf)*

*Re: Corrected L 334(deleted a in rbcl)*

**Line 344 when discussing such results, it would be highly desirable to refer to the percent plant coverage of each species since one large acacia tree** **may be more important as a food source than many small bushes or grasses or weeds. Try to** **discuss host preferance vs. host availability (exactly as we do when we discuss blood hosts)**

*Re:* *We have rewritten this part to read “Whether proclivity on Acacia is related to its biological traits, that is, a tree with a large biomass containing a range of nutrients, which may present it as a more important available and accessible food source than many small bushes or grasses or weeds to sandflies, is unclear. More research is needed to unravel the strong association with Acacia. L439-443*

*Re: Corrected L 354 (deleted) (……profiles of the Fabaceae showed that sand flies…)*

**Line 353 This table is superfluous within the text. summarize it and place the entire table in the supplementary data section**

*Re: This table has been revised and included as supplementary one (S1 Table). Removed from ms.*

**Line 376-377 what does ns stand for?**

*Re:* *Corrected (ns, not significant) L389*

*Re: Corrected L 404 (deleted profile)*

*Re: Corrected L 430 (ecological zone in Kenya)*

*Re: Corrected L 459 (reference)*

Reviewer #3: (No Response)

--------------------

<b>Summary and General Comments</b></br></br>
Use this section to provide overall comments, discuss strengths/weaknesses of the study, novelty, significance, general execution and scholarship. You may also include additional comments for the author, including concerns about dual publication, research ethics, or publication ethics. If requesting major revision, please articulate the new experiments that are needed.

**Reviewer #1: This is a well-designed study with clear public health significance.  I found it easy to follow and enjoyable to read.  The combination of analyses, including fructose positivity, DNA extractions and sequencing, and analyzing volatile organic compounds has furthered our understanding of the feeding behavior of this important vector. I think the study could be strengthened by including a survey of the relative abundance of the plant species found in the study site.**

*Re: Thanks (no response need). Good and important point but beyond the scope of the current study. A future study will consider to include this idea*

Reviewer #2: Good paper, nice work, worth publishing with some attention to detail and corrections as suggested in the annotated ms attached

*Re: Thanks (no response need)*

**Reviewer #3: Materials and Methods
132: How many CDC traps by habitats?**

*Re: This information has been provided in the text: Three-four traps were randomly deployed to collect night-active sand flies daily for eight consecutive days from three habitat types: houses indoors and outdoors, around termite mounds and animal sheds. L133-135*

**134: Please, for the first time to cite the Institute: use the abbreviation after the entire name: International Centre of Insect Physiology and Ecology (ICIPE). In the next times (lines 193 and 211) use only the abbreviation in capital letters (ICIPE).**

*Re: Corrected L136-137, 198, 211*

**214:** **20 mg of each of the ground plant samples (leaves) were extracted
224: 10 μl of each plant and sand fly sample extract were analyzed
Results
Considering the technique used to detect the sand fly host plants, is it possible to identify more than one plant in the gut of one individual? If the answer is positive, this event did not happen with the samples? I suggest a phrase related to this issue.**

*Re:* *Using the PCR employed, multiple bands indicative of different plants can be amplified from a single sample.* *We have included the following sentences in the method section viz: Among unsuccessful sequenced specimens were those (n=5) from which multiple DNA bands indicative of mixed plant meals (from different host plants) were detected. The data presented indicate plant DNA detection from single plant meals (one host plant) in individual samples. L335-339.*

**321: “Fructose positive rate of 44.7% (67/150) for females and 32.7% (49/150) for males significantly differ for collections indoors (χ2 = 2.8, df = 1, P < 0.09).” Considering the p value < 0.09, this is not statistically different.**

*Re: Corrected L322 (did not differ significantly)*

**Table 3. Is there any relevance for presenting the Sample ID and Accession? If not, I suggest that you remove both columns. I am curious to know which one of those specimens were collected indoor or outdoor. I suppose you could add this information in this Table.**

*Re: This table has been revised as suggested and included as a supplementary table (S1 Table). Removed from ms*

**Table 3. You should mention in text what happened with the 7 individuals that were not identified. Were they damaged? Also, the legend N/A should be changed for (-): not identified (or damaged)**

*Re: They were identified except that the common names are not known (three not seven). A footnote (-) indicating these samples with no known common names has been included. See S1 Table.*

**Tables 4 and 5: Please, verify the legends; ng/mg is it correct?**

*Re: the amount in ng/mg (mass/mass) is correct for both tables*

**Fig 4: The “non-metric multidimensional scaling plot (NMDS) clustering of the volatile
organic compounds during the (A) day and (B) night collection” are not with a good resolution. Is it possible to improve the quality of the image?**

*Re: We have modified this figure to improve on clarity. (Fig 4)*

**Moreover, they are not related in the text (results section). Only C and D are cited in the text.**
*Re: Fig 4A and 4B now cited in the text. L 405-406*

**Discussion

I suggest one paragraph about the leishmaniasis vectors species found in this study.**
*Re: This information has been included in the sentence.* *Here, we report the feeding patterns of wild-caught sand flies including P. martini and P. dubosqci vectors of visceral and cutaneous leishmaniasis, respectively, from a leishmaniasis endemic area in a dry ecological zone in Kenya. L428-430*

**430: acacia – Acacia (Please, revise the document and change all by capital letters).**
*Re: Corrected throughout the document*

**445: “Other adaptations than abundance may explain the seeming strong association between acacias and diverse sand fly species at the study site during the study period.” This phrase is too vague and also it is missing the reference.**

*Re: We have rewritten this part to read “Whether proclivity on Acacia is related to its biological traits, that is, a tree with a large biomass containing a range of nutrients, which may present it as a more important available and accessible food source than many small bushes or grasses or weeds to sandflies, is unclear. More research is needed to unravel the strong association with Acacia*

*(L439-443)*

**502: “While the data generated suggest selective sand fly feeding, the success rate of plant DNA detection including sequencing from fructose positive sandflies was low (<6%).” How did you find this percentage? Because if you considered 40 sequenced successfully by the total: 600, the result is 6,7%.**

*Re: This has been corrected to 6.7%. L514*

--------------------

PLOS authors have the option to publish the peer review history of their article (<a href="<https://journals.plos.org/plosntds/s/editorial-and-peer-review-process#loc-peer-review-history>" target="_blank">what does this mean?</a>). If published, this will include your full peer review and any attached files.<br><br>

If you choose “no”, your identity will remain anonymous but your review may still be made public.<br><br>

<b>Do you want your identity to be public for this peer review?</b> For information about this choice, including consent withdrawal, please see our <a href="<https://www.plos.org/privacy-policy>" target="_blank">Privacy Policy</a>.

Reviewer #1: Yes: Mark F. Olson

Reviewer #2: No

Reviewer #3: No
